# Erg currents support electrical bursting in murine anterior pituitary corticotrophs

**DOI:** 10.1113/JP290793

**Published:** 2026-05-11

**Authors:** Sooraj V. Nair, Nicola Romanò, Peter J. Duncan, Paul Le Tissier, Richard Bertram, Michael J. Shipston

**Affiliations:** ^1^ Institute for Neuroscience and Cardiovascular Research Edinburgh Medical School University of Edinburgh Edinburgh UK; ^2^ Department of Mathematics and Programs in Molecular Biophysics and Neuroscience Florida State University Tallahasee Florida USA

**Keywords:** anterior pituitary, corticotroph, Erg, hypothalamic‐pituitary‐adrenal axis, Kcnh2, potassium channel, stress

## Abstract

**Abstract:**

The regulation of electrical excitability of anterior pituitary corticotrophs is critical for an appropriate response of the hypothalamic‐pituitary‐adrenal (HPA) axis in the face of diverse physiological challenges in health and disease. However ion channels that control corticotroph excitability remain poorly characterised. Members of the mammalian ether‐à‐go‐go (EAG) channel family are voltage‐gated potassium channels with diverse functions in the endocrine, cardiovascular and nervous systems. Expression of *Kcnh2* mRNA, which encodes for the Eag‐related potassium channel Erg1, is enriched in the anterior pituitary although its functional role is poorly understood in native anterior pituitary cells.

We reveal that *Kcnh2* is the major Eag‐channel family member mRNA expressed in male and female murine corticotrophs. Patch clamp electrophysiological analysis revealed corticotrophs exhibit robust Erg‐like currents that are inhibited by the selective Erg‐inhibitor E4031 but are not regulated by the major hypothalamic secretagogues, corticotrophin releasing hormone (CRH) or arginine vasopressin (AVP), that increase corticotroph excitability. Pharmacological inhibition of Erg currents had no effect on spontaneous electrical excitability in corticotrophs. Rather, paradoxically, Erg currents were important for supporting CRH‐induced bursting, similar to the role of large‐conductance calcium‐ and voltage‐activated potassium (BK) channels, providing a level of redundancy to control bursting. Indeed mathematical modelling revealed that in the absence of BK channels CRH‐induced bursting can be supported in thepresence of Erg current.

We thus reveal a novel role for Erg‐like channels in controlling CRH‐induced bursting in murine anterior pituitary corticotrophs that is likely to be an important determinant of HPA axis regulation in health and disease.

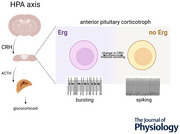

**Key points:**

- Voltage‐gated ether‐à‐go‐go‐related (Erg) potassium channels are key determinants of cellular excitability; however their functional role in native anterior pituitary cells remains poorly understood.
- Male and female murine corticotrophs predominantly express Kcnh2 (Erg1) mRNA, and electrophysiological recordings reveal functional Erg‐like potassium currents sensitive to the Erg inhibitor E4031.
- Erg currents do not control the basal electrical excitability of corticotrophs.
- We reveal a novel role for Erg currents in supporting electrical bursting in corticotrophs induced by the hypothalamic secretagogue, corticotrophin releasing hormone (CRH).
- Bursting in corticotrophs can be supported by both Erg and BK channels revealing a level of redundancy to the CRH response that will be critical for understanding the role of Erg channels in control of the stress axis in health and disease.

## Graphical abstract

**Abstract figure legend** Anterior pituitary corticotrophs are a central component of the hypothalamic‐pituitary‐adrenal (HPA) axis that controls the release of glucocorticoids from the adrenal gland in response to stress. The hypothalamic secretagogue corticotrophin releasing hormone (CRH) promotes electrical bursting of mouse anterior pituitary corticotrophs to release the stress hormone adrenocorticotrophin hormone (ACTH). Here we show that CRH‐induced electrical bursting is supported by Erg1 (Kcnh2) voltage‐gated potassium channels that are expressed in mouse corticotrophs. Inhibition of Erg1 attenuates CRH‐induced bursting, resulting in an increase in spiking induced by CRH. Our data define a novel role for Erg in supporting CRH‐induced bursting, a role also previously reported for calcium‐ and voltage‐activated potassium (BK) channels in corticotrophs, revealing a level of redundancy to the CRH response that will be critical for understanding the role of Erg channels in control of the stress axis in health and disease.

## Introduction

Mammalian ether‐à‐go‐go (EAG) channels encoded by three *Kcnh* gene subfamilies – *Eag* (*Kcnh1, Kcnh5*); Eag‐related, *Erg* (*Kcnh2, Kcnh6, Kcnh7*); and Eag‐like, *Elk* (*Kcnh3, Kcnh4, Kcnh8*) – are voltage‐gated potassium channels with diverse properties, expression patterns and physiological functions (Babcock & Li, 2013; Barros et al., 2020; Bauer & Schwarz, 2018; Sanchez‐Conde et al., 2022; Vandenberg et al., 2012). In particular the unusual gating kinetics of Erg1, encoded by *Kcnh2*, reveals a diverse array of functions across the cardiovascular, nervous and endocrine systems since its original identification as a key determinant in controlling cardiac action potential duration.

In many neurones and endocrine cells Erg channels contribute to the control of interspike interval and spike accommodation as the channels rapidly inactivate after depolarisation to positive voltages and then reactivate before closing during membrane repolarisation (Chiesa et al., 1997; Schonherr et al., 1999). Indeed pharmacological inhibition of Erg channels can promote basal and/or secretagogue‐evoked cell depolarisation and increase action potential firing in a variety of endocrine cells, including in the pituitary gland, adrenal gland and pancreas (Bauer, 1998; Bauer et al., 1999; Gullo et al., 2003; Hardy et al., 2009; Hirdes et al., 2010; Kirchberger et al., 2006; Lecchi et al., 2002; Rosati et al., 2000; Schafer et al., 1999; Storey et al., 2002, 2006) as well as different neurones (Bauer & Schwarz, 2018; Sanchez‐Conde et al., 2022). However their physiological role is cell type and context specific so that both hyperexcitability and hypoexcitability of different neurones or endocrine cells may be controlled depending on both cell intrinsic factors and intercellular signalling (Babcock & Li, 2013; Bauer & Schwarz, 2018; Hardy et al., 2009; Rosati et al., 2000; Sanchez‐Conde et al., 2022; Vandenberg et al., 2012).

*Kcnh2* (*Erg1*) mRNA expression is enriched in the anterior pituitary (Sanchez‐Conde et al., 2022); however the functional role in different native pituitary cell types remains poorly understood. Pharmacological inhibitors such as the type III anti‐arrhythmic methanesulfonanilide E4031 that inhibits all Erg, but not Eag currents (Herzberg et al., 1998; Shi et al., 1997), have revealed an important role for Erg‐like currents in the control of both native lactotroph and gonadotroph electrical excitability as well as in clonal lactotroph cell lines such as GH3/B6, MMQ and GH4C1 cells (Bauer, 1998; Bauer et al., 1999; Kirchberger et al., 2006; Lecchi et al., 2002; Schafer et al., 1999; Storey et al., 2002, 2006). Importantly in these systems hypothalamic neuropeptides that activate G‐protein coupled receptors (GPCR) coupled to the Gq/11 pathway can inhibit Erg currents to promote excitability. For example thyrotrophin releasing hormone (TRH) inhibits Erg‐like currents most likely via a variety of pathways in native and clonal lactotrophs. Furthermore circulating hormones such as thyroid hormone can activate Erg currents in GH4C1 lactotrophs to suppress excitability (Storey et al., 2002, 2006). Gonadotrophin‐releasing hormone (GnRH) also inhibits Erg‐like currents in murine gonadotrophs through its Gq/11 GPCR to enhance spike frequency (Hirdes et al., 2010).

However the role of Erg‐like currents in controlling the excitability of most native anterior pituitary cells is very poorly understood, especially in cells that exhibit extensive bursting behaviour such as corticotrophs. Bursting provides a mechanism for robust secretagogue‐induced secretion compared to single‐action potential spiking (Tagliavini et al., 2016). The excitability of anterior pituitary corticotrophs is regulated by two major hypothalamic neuropeptides, corticotrophin releasing hormone (CRH) and arginine vasopressin (AVP), that act via distinct GPCRs. In murine corticotrophs spontaneous electrical excitability is characterised by low‐frequency single‐action potentials. CRH activates the CRHR1 GPCR to stimulate cAMP production that results in a transition from spontaneous single‐action potential spiking to pseudoplateau bursting (Duncan et al., 2015; Duncan et al., 2023; Duncan et al., 2024; Zemkova et al., 2016). In contrast AVP through the AVPR1b GPCR activates the Gq pathway to stimulate an increase in spike frequency (Duncan et al., 2015; Duncan et al., 2023; Duncan et al., 2024; Zemkova et al., 2016). Although a potential role for Erg‐like currents in rat corticotroph function has been described (Yamashita et al., 2009), whether corticotrophs express functional Erg‐like currents and whether Erg channels are an important determinant of corticotroph excitability are unknown.

Here we address whether murine corticotrophs express functional Erg‐like currents and examine the role of Erg‐like currents in both spontaneous and secretagogue‐evoked excitability. We reveal a novel and apparent paradoxical role for Erg‐like currents in supporting bursting behaviour in corticotrophs in contrast to the previously reported role in limiting spike frequency in lactotrophs and gonadotrophs.

## Methods

### Ethical approval

All breeding and tissue collection were performed in accordance with UK Home Office requirements (PPL PP2870833) and University of Edinburgh Ethical Review Committee approval (PL26‐21).

Mice expressing green fluorescent protein (GFP) under the proopiomelanocortin (POMC) promoter were used as previously described (Duncan et al., 2015) on a C57/BL6 background. Mice were caged in groups of two to four under standard laboratory conditions (lights turned on at 7:00 AM, lights turned off at 7.00 PM, 21°C, with tap water and chow available *ad libitum*) at the University of Edinburgh. Male and female mice aged 2–4 months were used for pituitary cell culture from tissue collected between 8:30 and 10:00 AM after cervical dislocation.

### Reagents

General biochemical reagents used throughout this study were obtained from Sigma‐Aldrich (St. Louis, MO, USA) and were of analytical‐grade quality unless stated otherwise. CRH and [Arg^8^]‐vasopressin (AVP) acetate salt were obtained from Bachem AG (Bubendorf, Switzerland). Dofetilide (DOF, UK‐68798) was obtained from Selleckchem (Houston, TX, USA). E‐4031 was obtained from Biotechne/TOCRIS (Abingdon, UK).

### Primary corticotroph cell culture

Anterior pituitary cells were acutely isolated by trypsin digestion as previously described (Duncan et al., 2015). Typically each cell preparation used anterior pituitary glands collected from three animals. For electrophysiological experiments cells were cultured on 12 mm coverslips (Warner Instruments, Holliston, MA, USA) in serum‐free medium (Dulbecco's modified Eagle's medium containing 25 mm HEPES, 5 µg/mL of insulin, 50 µg/mL of transferrin, 30 nm sodium selenite, 0.3% bovine serum albumin (BSA, w/v), 4.2 µg/mL of fibronectin and antibiotic/antimycotic (100× dilution of Sigma stock)) and incubated at 37°C in 5% CO_2_. Serum‐free medium (lacking antibiotic/antimycotic) was changed every 2 days, and electrophysiological recordings were obtained from cells 24–96 h postisolation.

### Electrophysiology

Electrophysiological recordings were obtained from GFP‐identified corticotroph cells using the perforated patch mode of the whole‐cell patch clamp technique. Amphotericin B was used at a concentration of 150 µg/mL in pipette solution, which resulted in compensated access resistances typically <20 MΩ within 10–20 min and allowed stable recordings for >40 min. Coverslips were housed in a recording chamber situated on an Eclipse TE200 Inverted fluorescent microscope (Nikon). Recordings were obtained using an Axopatch 200B amplifier (Axon Instruments) coupled with a Digidata 1320A A/D converter (Axon Instruments) and linked to a computer. Recordings were performed at room temperature (18–22°C) with protocols and acquisition using Clampex 9.0 or 10.7 (Molecular Devices, San Jose, CA, USA) with a sampling rate of 10 kHz and filtered at 2 kHz. Patch pipettes were manufactured from borosilicate glass (King Precision Glass, Inc., Claremont, CA, USA) using a model P‐97 micropipette puller (Sutter Instrument Co., Novato, CA, USA). Pipette tips were heat polished and had resistances typically between 2 and 3 MΩ. Capacitance of corticotrophs ranged from 2 to 10 pF. A gravity‐driven perfusion system was used to apply drugs to the cells with a flow rate of 1–2 mL/min to minimise flow‐induced artifacts.

To isolate and characterise Erg‐like currents by voltage clamp, the perforated patch clamp mode was used with a high K^+^ bath solution (extracellular), which helps to delay the inactivation process and also increases the unitary conductance (Vandenberg et al., 2012). The bath solution contained 140 mm KCl, 0.1 mm CaCl_2_, 2 mm MgCl_2_, 10 mm HEPES and 10 mm glucose, at pH 7.4, and the pipette (intracellular) contained 110 mm K‐gluconate, 35 mm KCl, 2 mm MgCl_2_, 1 mm CaCl_2_ and 10 mm HEPES at pH 7.4. For biophysical analysis of Erg‐like currents, two biophysical pulse protocols were employed (Storey et al., 2002, 2006). Firstly to determine the time course of CRH+AVP effects on currents, a ‘single‐step’ protocol was applied every 30 s from a holding potential of −40 mV, then stepping up to +40 mV for 1 s, then reverting to −40 mV for 100 ms and then measuring the peak current at a hyperpolarising potential of −120 mV for 1 s. Secondly to determine the ‘steady‐state activation’ and current–voltage relationship, a multistep protocol was performed (Storey et al., 2002, 2006) from a holding potential of −40 mV for 100 ms to a step of 4‐s duration to −80 to + 70 mV in 10 mV increments before stepping up to −40 mV for 100 ms and measuring the peak current in response to a hyperpolarising pulse to −100 mV for 2 s to activate Erg channels. Cells were then stepped up to −40 mV for 800 ms as holding potential between sweeps.

Activation curve data from individual cells were best fit to a Boltzmann function of the form:


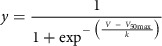

where *V*_50max_ is the voltage for half‐maximal activation and *k* is the slope factor describing the steepness of voltage sensitivity. Group means and standard deviations were determined by averaging *V*_50max_ and *k* values from across multiple cells in Prism.

For currents recorded during time course analysis using the single‐step pulse protocol to determine currents at −120 mV from a step of 40 mV, the time constants of channel kinetics were determined by a best fit of a double exponential in Clampfit for both fast (τ_fast1_ and τ_fast2_) and slow (τ_slow1_ and τ_slow2_) current kinetics.

For current clamp analysis the perforated patch clamp technique was used for the electrophysiological recordings in physiological ion gradients. The bath solution was filled with a standard bath (extracellular) solution containing 140 mm NaCl, 5 mm KCl, 2 mm CaCl_2_, 0.1 mm MgCl_2_, 10 mm HEPES and 10 mm glucose. The pH and osmolality were adjusted to 7.4 with NaOH and 300 mOsmol/L, respectively. The standard pipette solution (intracellular) contained the following (in mM): 10 NaCl, 30 KCl, 60 K_2_SO_4_, 1 MgCl_2_, 10 HEPES, 10 glucose and 50 sucrose. The pH and osmolality were adjusted to 7.3 with KOH and 290 mOsmol/L, respectively.

### Electrophysiological analysis of excitability

Current clamp recordings were analysed as previously described (Duncan et al., 2015; Duncan et al., 2023) using Clampfit, version 10.7 (Molecular Devices). Corticotroph excitability was measured for 3 min under basal conditions and during secretagogue‐evoked activity or exposure to Erg inhibitors. Membrane potential was calculated by averaging three time points at the beginning, middle and end of the measurement period. Properties of spikes and bursts (collectively ‘events’) were measured using the Event Detection function of Clampfit software and manually verified. An event was defined as the period from which the point membrane potential reached the threshold (Δ20 mV from baseline) until it decreased below a re‐arm level (Δ5 mV). This allowed the separation and analysis of properties of single spikes, as well as bursts with calculation of frequency or duration of any event classified as a spike or burst. This method classifies any event <100 ms in duration as a spike and events >100 ms in duration, which also have at least two spikelets during the event, as a burst. Bursting behaviour was quantified by calculating a burst factor (BF), obtained as the proportion of the number of total events that are bursts (Duncan et al., 2015; Duncan et al., 2023; Tabak et al., 2011).

### Computer simulations

We used a published model of electrical activity in corticotrophs (Duncan et al., 2015), with the addition of an Erg‐like current to perform computer simulations that examine how it might contribute to bursting activity. The corticotroph model is described in detail in Duncan et al. (2015), so we focus on important elements and the Erg current model.

The model contains two types of BK currents, those responding to the Ca^2+^ concentration in the domain at the mouth of open Ca^2+^ channels (BK_near_ channels) and those further from Ca^2+^ channels and gated by the bulk cytosolic Ca^2+^ concentration (BK_far_ channels). We showed in Duncan et al. (2015) and Fazli et al. (2021) that the BK_near_ channels are key for bursting. We include both populations of BK channels in the model, though in some simulations the BK_near_ channels are removed. Application of CRH is simulated as in Duncan et al. (2015). The conductance of L‐type Ca^2+^ channels ($g_{\mathrm{Ca}}$) is increased from 1.8 to 2.4 nS, the value of BK_near_ channel half‐activation is modified by increasing $k_{\mathrm{Ca}-\mathrm{BK}-\mathrm{near}}$ from 2 to 6 µM and the BK_near_ time constant is reduced from $\tau_{\mathrm{BKn}}=20$ to 3 ms.

The Erg‐like current is modelled as a K^+^ current with both activation and inactivation. The current is

(1) $$I_{\mathrm{Kerg}}=g_{\mathrm{Kerg}}ad\left(V-V_{K}\right)$$

with conductance $g_{\mathrm{Kerg}}$, Nernst potential $V_{K}=-75\;\mathrm{mV}$, activation variable *a* and inactivation variable *d*. The differential equations for the activation/inactivation variables are

(2) $$\frac{da}{dt}=\frac{a_{\infty }\left(V\right)-a}{\tau_{a}}\;$$

(3) $$\frac{dd}{dt}=\frac{d_{\infty }\left(V\right)-d}{\tau_{d}}\;$$

where the equilibrium functions are

(4) $$\;a_{\infty }\;\left(V\right)={\left[1+\exp\left(\frac{v_{a}-V}{s_{a}}\right)\right]}^{-1}\;$$

(5) $$\;d_{\infty }\;\left(V\right)={\left[1+\exp\left(\frac{v_{d}-V}{s_{d}}\right)\right]}^{-1}\;$$

The activation time constant, $\tau_{a}=100\;\mathrm{ms}$, is much larger than that for inactivation, $\tau_{d}=10\;\mathrm{ms}$. The half‐activation voltage is $v_{a}=-25\;\mathrm{mV}$, and the half‐inactivation voltage is $v_{d}=-10\;\mathrm{mV}$. The slope parameters have equal magnitudes, $s_{a}=10\;\mathrm{mV}$ and $s_{d}=-10\;\mathrm{mV}$.

The two non‐BK type K^+^ currents in the model are the delayed rectifier current, *I*_Kdr_, and the Erg current, *I*_Kerg_. Both have maximum conductances equal to 8 nS. These are then adjusted by a parameter β∈[0,1] as follows:

(6) $$g_{\mathrm{Kerg}}=\beta g_{\mathrm{Kerg}\_\max}$$

(7) $$g_{\mathrm{Kdr}}=\left(1-\beta \right)g_{\mathrm{Kdr}\_\max}$$

The parameter β is used to vary the contribution that Erg current makes relative to the delayed rectifier, and the default value is β=0.5. In simulations in which Erg current is partially inhibited we used β=0.25.

The free software package XPPAUT was used in computer simulations, and the computer code can be downloaded from https://www.math.fsu.edu/~bertram/software/pituitary and from https://github.com/rbertram.

### Data analysis

Data in the text are presented as mean ± SD, where *n* represents the number of cells from across at least independent preparations of cells, with each preparation normally generated from three animals. Data in figures are presented as box plots divided into quartiles and overlaid with data points from individual cells. Statistical analyses of electrophysiological parameters were performed using Prism 10. Quantitative data were analysed using linear mixed‐effects models (lme) for paired data where analysis compared effects in the same cell (e.g. effect of E4031 on spontaneous excitability, effect of CRH+AVP) or unpaired data for comparisons between different populations (e.g. between sexes, between different cells pretreated with either control or drug). *Post hoc* comparisons were performed using Tukey's test when main effects or interactions were found to be significant. Significant differences between groups were defined for *P* < 0.05 and are represented to three significant figures.

## Results

### Kcnh2 is the dominant Kcnh gene mRNA expressed in murine corticotrophs

**Figure 1:**
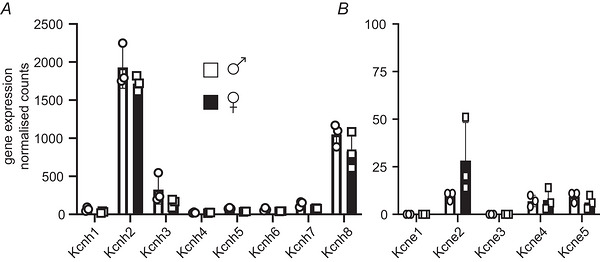
*Kcnh2* (*Erg1*) is the major EAG (ether‐à‐go‐go)‐family mRNA expressed in mouse corticotrophs Bar chart of *A*, EAG‐family (*Kcnh*) pore‐forming subunit and *B*, accessory subunit (*Kcne* gene) gene expression from RNA‐seq analysis of purified male (open box) and female (filled box) mouse anterior pituitary corticotrophs. mRNA gene expression is expressed as normalised counts (mean ± SD) from 3 independent RNA‐seq experiments in both sexes.

We first analysed published RNA‐seq data from purified murine male and female corticotrophs (Duncan et al., 2023; Duncan et al., 2024) to determine the relative mRNA expression of the Eag‐family members *Kcnh1–Kcnh8*. *Kcnh2*, which encodes for mouse Erg1, was the most abundant mRNA in both male and female corticotrophs with an expression at least an order of magnitude higher than most other detectable *Kcnh* subunit mRNAs (Fig. 1*A*). *Kcnh8 m*RNA, which encodes for mouse Elk1, was also expressed in both male and female corticotrophs at mRNA levels approximately half that of *Kcnh2*. No significant differences in mRNA expression for any subunit were observed between male and female corticotrophs (Fig. 1*A*). As *Kcnh2* is the dominantly expressed *Kcnh* mRNA, we also examined the mRNA expression of the primary accessory subunits for *Kcnh*2. Kcne2 (MiRP1) and Kcne1 (MinK) are the major accessory subunits of Kcnh2, and related subfamily members may also play a role. *Kcne2 *mRNA was expressed at low levels, but other *Kcne* subunit mRNAs were at, or below, the limit of detection (Fig. 1*B*).

### Murine corticotrophs express E4031‐sensitive ionic currents

**Figure 2:**
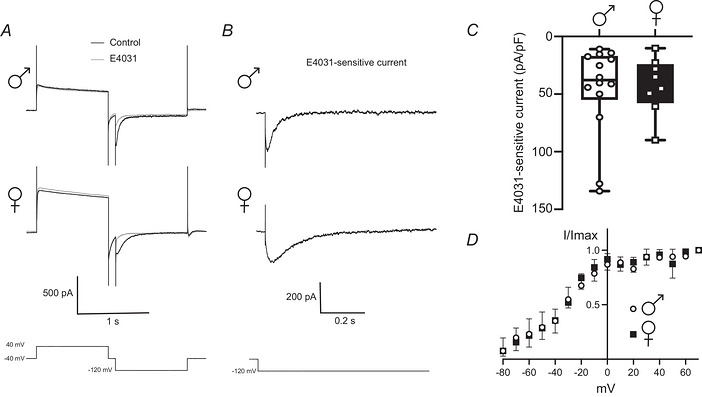
Murine corticotrophs express functional E4031‐sensitive currents *A*, exemplar traces of fast and slow tail current kinetics observed in both male and female murine corticotrophs; representative traces are shown from a male and female corticotroph, respectively, during a control period (black line) and after the addition of 5 µm E4031 (grey line) to the same cell. A step protocol for biophysical isolation of Erg‐like currents was used to isolate an inward current at the step from −40 to −120 mV in perforated patch clamp recordings. *B*, E4031‐sensitive currents are plotted from the same male and female corticotroph as in panel *A* determined by subtracting the current in the presence of E4031 from the control current in panels *A* and *B*, respectively, and currents exhibited at higher resolution. *C*, box and whisker plot of E4031‐sensitive current density (pA/pF) in male (open box, *n* = 14) and female (filled box, *n* = 8) from traces as in panel *B*. *D*, current–voltage relationship for E4031‐sensitive currents in male (open box) and female (filled box) corticotrophs using a multistep protocol as described in ‘Methods’ section across a range of potentials. Data are expressed as mean ± SD; *n* = 9 and 4 cells for males and females, respectively.

As *Kcnh2* (*Erg1*) was the most highly expressed Eag‐family subunit mRNA, and relatively specific pharmacological inhibitors are available, we initially characterised Erg‐like currents in murine corticotrophs using the perforated patch clamp recording configuration in voltage clamp. To characterise Erg‐like currents we exploited both a biophysical and pharmacological isolation approach. In initial experiments we used a repeated voltage protocol to determine the Erg‐like peak tail currents determined at −120 mV in the presence and absence of 5 µM of E4031 applied to the bath solution (Fig. 2*A*). Under these conditions E4031‐sensitive currents could be readily isolated in both male and female corticotrophs (Fig. 2*B*). However E4031‐sensitive current density was highly heterogenous in both males and females with almost an order of magnitude difference in current density between cells. We also observed differences in the kinetics of the currents, with both fast and slow current kinetics observed in both male and female corticotrophs (Fig. 2*A, B *and* C*). To determine the kinetics in detail, we analysed the time constant of the tail currents at −120 mV that were best fit with a double exponential resulting in two time constants τ_fast1_ and τ_fast2_. Currents with fast kinetics were observed in 6 of 14 male cells, with τ_fast1_ and τ_fast2_ of 19.01 ± 3.01 and 66.62 ± 4.63 ms, respectively. Cells with slow current kinetics (8 of 14 cells) had significantly longer τ_slow1_ and τ_slow2_ of 48.78 ± 10.15 ms (*P* < 0.0001, compared to τ_fast1_) and 206.78 ± 26.35 ms (*P* < 0.0001 compared to τ_fast2_), respectively, compared to cells with fast kinetics. In females 3 of 8 cells exhibited fast current kinetics and 5 of 8 cells slow current kinetics, with time constants not significantly different from the respective time constants in males: τ_fast1_ = 19.34 ± 5.68 ms and τ_fast2 = _66.91 ± 6.24 ms; τ_slow1_ = 47.32 ± 10.23 ms and τ_slow2 = _199.51 ± 16.93 ms.

**Figure 3:**
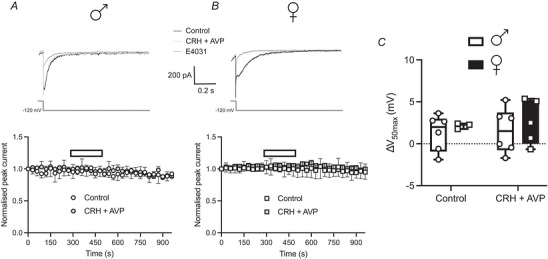
CRH (corticotrophin releasing hormone) and AVP (arginine vasopressin) do not regulate E4031‐sensitive currents in murine corticotrophs Representative current traces of biophysically isolated Erg‐like currents as in Fig. 2*B* from a voltage step from −40 to −120 mV and time course of normalised peak current in *A*, males and *B*, females. Current traces show overlaid currents in control (black line), in the presence of 0.2 nm CRH and 2 nm AVP (light grey) and after exposure to E4031 (dark grey). In the lower time course panels, the horizontal open bar indicates the period of application of CRH+AVP or vehicle control. Data are expressed as normalised peak current (mean ± SD) in control (open box) and after CRH+AVP (light‐grey box). In panel *A* male cells *n* = 4 and 6 for control and CRH+AVP‐treated cells, respectively. In panel *B* female cells *n* = 5 and 7 for control and CRH+AVP‐treated cells, respectively. *C*, box and whisker plots of the change in voltage for half‐maximal activation (Δ*V*_50max_), determined as in Fig. 2*D*, before and after the vehicle control or application of CRH+AVP in male (open box, *n* = 6 and 6 for control and CRH+AVP, respectively) and female (filled box, *n* = 3 and 5 for control and CRH+AVP, respectively) murine corticotrophs.

There was no significant difference in mean current density between male and female corticotrophs (Fig. 2*C*), with male current density −46.2 ± 49.6 pA/pF (range: −11 to −134 pA/pF) and female current density −42.6 ± 29.0 pA/pF (range: −10.1 to 90 pA/pF). In addition to the heterogeneity in E4031‐sensitive current density, the proportion of peak biophysically isolated current that was E4031 sensitive varied widely. In the majority of cells greater than 90% of the biophysically isolated peak current was inhibited by E4031, although in a small number of cells E4031 inhibited only 60% of the current. Under control conditions the biophysically isolated currents were stable over a period >20 min under the recording configuration used (see Fig. 3*A *and* B*).

To further characterise the E4031‐sensitive currents, we used a multistep protocol to construct current–voltage relationships for the E4031‐sensitive currents to determine the voltage for half‐maximal activation (*V*_50max_; Fig. 2*D*). The pharmacologically isolated E4031‐sensitive current in males and females had a similar *V*_50max_ of −37.41 ± 9.63 mV, *n* = 9, and −34.38 ± 3.62 mV, *n* = 4, respectively. The slope factor, *k*, for the E4031‐sensitive current was not different between sexes, with a value of 7.93 ± 0.35 (*n* = 9) and 8.49 ± 2.29 (*n* = 4) for male and female corticotrophs, respectively. Together these properties are characteristic of Erg currents recorded under the extracellular K^+^ recording conditions used (Bauer & Schwarz, 2018; Sanchez‐Conde et al., 2022).

### CRH/AVP does not regulate Erg‐like currents in murine corticotrophs

As Erg‐like currents are regulated by GPCR in anterior pituitary, as well as other excitable cells (Bauer, 1998; Bauer et al., 1999; Kirchberger et al., 2006; Lecchi et al., 2002; Schafer et al., 1999; Storey et al., 2002, 2006), we asked whether the Erg‐like currents were regulated by the major hypothalamic neuropeptides CRH and AVP that control corticotroph excitability in response to stressors. GPCR‐mediated inhibition of Erg currents is typically characterised by a decrease in maximum current amplitude as well as a right shift in the *V*_50max_ to more depolarised potentials (Bauer & Schwarz, 2018; Hirdes et al., 2010; Storey et al., 2002). We used the repeated single‐step protocol to determine the time course of Erg‐like current regulation during combined bath application of a 3‐min pulse of 0.2 nm CRH and 2 nm AVP (CRH+AVP), approximating levels to which corticotrophs are likely exposed to during a typical stressor. However in both male and female cells with significant E4031 currents, CRH+AVP had no significant effect on Erg‐like current amplitude during or after the CRH+AVP pulse compared to pre‐exposure control currents in the same cell (Fig. 3*A, B *and* C*). Under these recording conditions control currents were stable over the entire recording period. In cells subject to the multistep potential protocol to allow current–voltage relationships to be determined, there was also no significant change in *V*_50max_ after exposure to CRH+AVP in the same cell (Fig. 3*C*). In male corticotrophs the change in *V*_50max_ after CRH+AVP was 1.55 ± 2.67 mV, *n* = 6, which was not different from that seen in cells exposed to vehicle control (1.31 ± 2.16 mV, *n* = 6). Similarly there was no significant change in *V*_50max_ after CRH+AVP in the same female corticotrophs, 2.59 ± 2.65 mV, *n* = 5, compared to the change in vehicle control (2.14 ± 0.34 mV, *n* = 3). In addition CRH+AVP had no significant effect on the slope factor. In male corticotrophs the change in slope factor after CRH+AVP exposure was 0.03 ± 2.18, *n* = 6, compared to the control slope factor before exposure in the same cell, and in female corticotrophs the change in slope factor was 0.28 ± 1.68, *n* = 5. Taken together these data reveal that CRH+AVP had no significant effects on Erg‐like currents over the time course in which CRH+AVP significantly increases corticotroph excitability.

### Inhibition of Erg‐like currents suppresses secretagogue‐induced bursting but has no effect on spontaneous electrical activity

In other endocrine cells, including anterior pituitary cells, pharmacological inhibition of Erg‐like currents has been reported to depolarise membrane potential and enhance spontaneous action potential frequency (Bauer, 1998; Bauer et al., 1999; Gullo et al., 2003; Hardy et al., 2009; Hirdes et al., 2010; Kirchberger et al., 2006; Lecchi et al., 2002; Rosati et al., 2000; Schafer et al., 1999; Storey et al., 2002, 2006). We thus first examined the effect of E4031 on spontaneous electrical excitability of male corticotrophs using the perforated patch clamp mode in physiological ion gradients. We first analysed the effect of bath application of E4031 on resting membrane potential, BF (a measure of the proportion of bursts to spikes with a low BF representing predominantly single spike activity), along with event frequency and event duration in male corticotrophs, where events include both spikes and bursts. Spontaneous electrical activity parameters were determined in control and during 10 min of bath application of E4031, a time window in which E4031 fully suppresses Erg‐like currents. The mean resting membrane potential of male corticotrophs during exposure to E4031 in the same cell was not significantly different from vehicle control. Membrane potential in the presence of E4031 was −50.25 ± 4.21 mV, *n* = 27, compared to control −50.34 ± 5.19 mV, *n* = 22. Furthermore E4031 had no significant effect on BF (0.04 ± 0.08 in the presence of E4031 compared to 0.11 ± 0.18 on control), event frequency (0.68 ± 0.73 Hz in the presence of E4031 compared with control cells 0.52 ± 0.68 Hz) or event duration (35.67 ± 33.35 ms in the presence of E4031 compared to control 60.59 ± 68.89 ms). In female corticotrophs mean resting membrane potential was −45.01 ± 2.92 mV, *n* = 5, and not significantly different compared to males, as previously reported (Duncan et al, 2023). E4031 had no significant effect on resting membrane potential compared to the control period in female cells: resting membrane potential was –44.47 ± 2.14 mV after E4031. As for male corticotrophs E4031 also had no effect on other spontaneous excitability parameters in female corticotrophs: basal BF was 0.13 ± 0.12 before E4031 addition and 0.21 ± 0.17 after E4031; spontaneous event frequency was 1.98 ± 1.42 Hz prior to E4031 addition and 1.75 ± 1.31 Hz after E4031; and spontaneous event duration was 64.05 ± 29.22 ms before and 72.83 ± 22.25 ms after E4031 exposure.

Thus in contrast to some other anterior pituitary cell types (Bauer, 1998; Bauer et al., 1999; Hirdes et al., 2010; Kirchberger et al., 2006; Lecchi et al., 2002; Schafer et al., 1999; Storey et al., 2002, 2006), pharmacological inhibition of Erg‐like currents with E4031 had no significant effect on spontaneous electrical behaviour of male or female murine corticotrophs.

**Figure 4:**
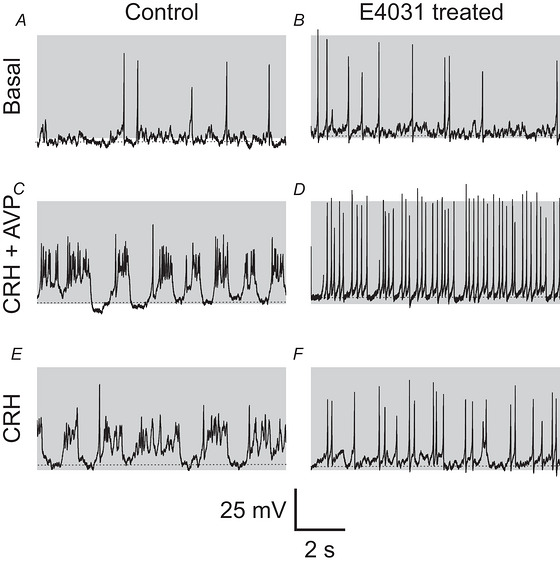
Pharmacological inhibition of Erg blunts CRH (corticotrophin releasing hormone)‐evoked bursting in corticotrophs Representative traces from independent cells of current clamp recordings of male corticotrophs in control cells or cells treated with E4031 under basal conditions (A & B respectively) and in response to a 3‐min exposure to 0.2 nm CRH and 2 nm AVP (arginine vasopressin) (C & D respectively), or 0.2 nm CRH alone (E & F respectively). Dotted line indicates resting membrane potential. Grey shading indicates membrane potential between −50 and +10 mV.

Although CRH+AVP had no effect on Erg‐like currents, we next asked if pharmacological inhibition of the Erg‐like currents modifies CRH+AVP‐induced excitability in corticotrophs. To address this we analysed the effect of CRH+AVP in the presence of either E4031 or another Erg inhibitor DOF in the perforated patch clamp configuration. E4031 inhibits all Erg channels, whereas DOF at this concentration also inhibits Eag channels (Babcock & Li, 2013; Barros et al., 2020; Bauer & Schwarz, 2018; Sanchez‐Conde et al., 2022; Vandenberg et al., 2012). We focused our analysis on male corticotrophs as they exhibit a much more consistent response to CRH, or AVP, than female corticotrophs (Duncan et al., 2023). In particular CRH evokes pseudoplateau bursting characterised by periods of depolarisation with low‐amplitude spikelets in the majority of male corticotrophs, whereas AVP predominantly induces an increase in spike frequency (Duncan et al 2015; Duncan et al., 2023). Bursting behaviour is less robust in a population of female corticotrophs (Duncan et al., 2023). In the current experiments cells were pretreated with either E4031 or DOF for 10 min prior to application of a 3‐min pulse of 0.2 nM CRH and 2 nM AVP, with activity determined for 3 min before and after the exposure to CRH+AVP. In control cells CRH+AVP results in membrane depolarisation and an increase in both spiking and bursting behaviour (Duncan et al., 2015; Duncan et al., 2022; Zemkova et al., 2016) as shown in Fig. 4.

**Figure 5:**
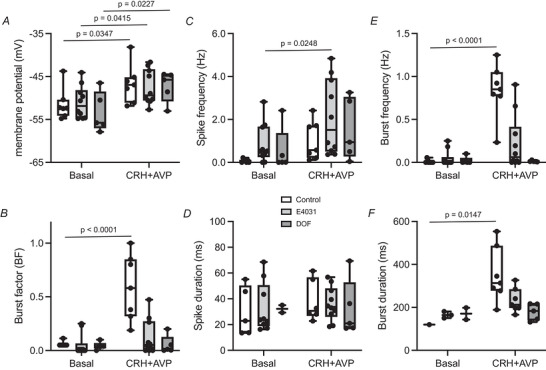
E4031 and dofetilide (DOF) inhibit bursting induced by CRH (corticotrophin releasing hormone) and AVP (arginine vasopressin) in corticotrophs Quantification of the effect of the Erg inhibitors E4031 (light‐grey box) and DOF (dark‐grey box) on basal and CRH+AVP (0.2 and 2 nM, respectively) induced electrical properties in male corticotrophs compared to control (open box) from current clamp recordings as in Fig. 4. *A*, membrane potential, CRH+AVP significantly increased membrane depolarisation in control and E4031‐ and DOF‐treated cells (*P* = 0.0347, 0.0415 and 0.0227, respectively); *B*, burst factor (BF) was significantly (*P* < 0.0001) increased by CRH+AVP in control cells; *C*, spike frequency was significantly increased (*P* = 0.0248) by CRH+AVP in E4031‐treated cells; *D*, spike duration was not significantly changed by CRH+AVP in any condition; *E*, burst frequency was significantly (*P* < 0.0001) increased by CRH+AVP in control cells; and *F*, burst duration was significantly increased (*P* = 0.0148) by CRH+AVP in control cells. Data are presented as box and whisker plots. *n* = 7, 10 and 5 cells for control, E4031 and dofetilide, respectively.

There was no significant difference in resting membrane potential between vehicle control‐, E4031‐ or DOF‐treated corticotrophs (Figs 4 and 5*A*) with a mean ± SD of −51.45 ± 3.69 mV (*n* = 7), −51.05 ± 3.78 mV (*n* = 10) and −53.37 ± 4.73 mV (*n* = 5), respectively (Fig. 5*A*). In all (7/7) control corticotrophs application of CRH+AVP resulted in a small but significant depolarisation of the membrane potential by 4.72 ± 1.52 mV (*n* = 7, *P* = 0.0347) that was not significantly different from the depolarisation induced by CRH+AVP in the presence of E4031 (3.48 ± 2.16 mV, *n* = 10) or DOF (6.06 ± 5.27 mV, *n* = 5) in all cells. This suggests that Erg‐like currents are not involved in CRH+AVP‐induced depolarisation of the membrane potential *per se* in agreement with the lack of effect of E4031 or DOF on resting membrane potential.

In control cells the response to CRH+AVP is characterised by transition from spiking to bursting reflected by an increase in BF, the proportion of events that are bursts *versus* spikes, that is predominantly driven by CRH (Duncan et al., 2015; Duncan et al., 2022; Zemkova et al., 2016) and an increase in spiking driven by AVP. In control cells spontaneous BF was low (0.06 ± 0.02, *n* = 7) and not significantly different from that in the presence of E4031 (0.05 ± 0.10, *n* = 10) or DOF (0.04 ± 0.04, *n* = 5) (Fig. 5*B*). In all seven control cells CRH+AVP significantly increased BF (to a mean ± SD of 0.59 ± 0.31, *n* = 7, *P *< 0.0001, generalized linear model (GLM), using Tukey's *post hoc* test). Although in 6 of 10 and 3 of 5 cells pretreated with E4031 or DOF, respectively, a small increase was observed in BF, the effect was not significantly different from spontaneous BF in E4031‐ (*P* = 0.3064) or DOF‐treated (*P* = 0.9209) cells (Fig. 5*B*). BF after CRH+AVP was 0.12 ± 0.16, *n* = 10, and 0.05 ± 0.08, *n* = 5, in the presence of E4031 and DOF, respectively.

However the lack of bursting was not a result of a lack of increased activity in corticotrophs as, in agreement with the similar depolarisation induced by CRH+AVP in the presence or absence of E4031 or DOF (Fig. 5*A*), CRH+AVP induced a significant increase in spike frequency in 9 of 10 E4031‐treated cells (from 0.86 ± 0.91 to 2.10 ± 1.75 Hz, *n* = 10, *P* = 0.0248 GLM using Tukey's *post hoc* test) (Fig. 5*C*). Although CRH+AVP induced an increase in spike frequency in all seven control and all five DOF‐treated cells, this did not reach statistical significance (*P* = 0.1810 and 0.2500, respectively). CRH+AVP had no significant effect on spike duration between any groups; similarly E4031 or DOF had no significant effect on spike duration compared to control cells (Fig. 5*D*).

The CRH+AVP‐induced increase in BF in control cells was associated with a significant increase in both burst frequency (Fig. 5*E*: from 0.01 ± 0.02 to 0.84 ± 0.32 Hz, *n* = 7, *P* < 0.0001) and burst duration (Fig. 5*F*: from 119.79 ± 0.01 to 350.99 ± 126.84 ms, *P* < 0.0148). CRH+AVP had no significant effect on burst frequency or burst duration in E4031‐ or DOF‐treated cells. Spontaneous burst frequency in E4031 and DOF was 0.05 ± 0.09 Hz, *n* = 10, and 0.02 ± 0.04 Hz, *n* = 5, respectively, whereas in thepresence of CRH+AVP it was 0.22 ± 0.32 Hz, *n* = 10, and 0.01 ± 0.01 Hz, *n* = 5, respectively. Spontaneous burst duration in E4031 and DOF was 164.99 ± 15.48 ms, *n* = 10, and 170.68 ± 38.88 ms, *n* = 5, respectively, whereas in the presence of CRH+AVP it was 232.10 ± 56.36 ms, *n* = 10, and 178.49 ± 35.12 ms, *n* = 5, respectively.

Taken together these data suggest that Erg‐like currents normally facilitate CRH+AVP‐induced bursting in male corticotrophs and that inhibition of these currents specifically limits bursting rather than the initial membrane depolarisation or change in spike frequency induced by CRH+AVP.

### E4031‐sensitive currents support CRH‐induced bursting

**Figure 6:**
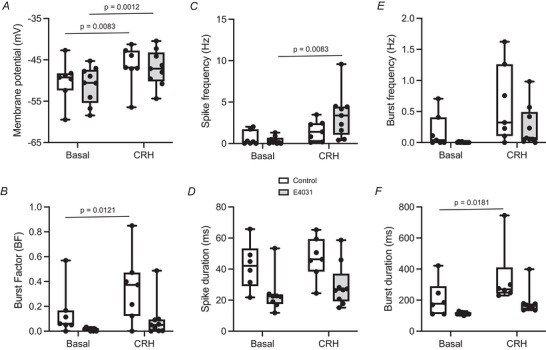
E4031 inhibits CRH (corticotrophin releasing hormone)–induced bursting in corticotrophs Quantification of the effect of the Erg inhibitor E4031 (light‐grey box) on basal and CRH (0.2 nM)–induced electrical properties in male corticotrophs compared to control (open box) from current clamp recordings as in Fig. 4 *. A*, membrane potential was significantly depolarised by CRH in both control and E4031‐treated cells (*P* = 0.0083 and 0.0012, respectively); *B*, burst factor (BF) was significantly increased (*P* = 0.0121) by CRH in control cells; *C*, spike frequency was increased significantly by CRH only in E4031‐treated cells (*P* = 0.0083); *D*, spike duration was not affected by CRH in either condition; *E*, although burst frequency was increased in all control cells, this did not reach significance (*P* = 0.0778); and *F*, burst duration was increased by CRH significantly only in control cells (*P* = 0.0181). Data are presented as box and whisker plots. *n* = 7 and 9 cells for control and E4031‐treated cells, respectively.

As CRH is the major driver of pseudoplateau bursting in mouse corticotrophs, and the effect of pharmacological inhibition was primarily on secretagogue‐induced bursting, we thus analysed the effect of pharmacological blockade of Erg‐like currents by E4031 on excitability induced by CRH alone. In all seven control cells CRH induced a small depolarisation of the membrane potential (Figs 4 and 6*A*) of 3.57 ± 1.93 mV, *n* = 7, that was not significantly different from that observed by CRH in E4031‐treated cells (4.05 ± 2.64 mV, *n* = 9). CRH‐induced a significant increase in BF from 0.17 ± 0.20 to 0.41 ± 0.25 (*n* = 7, *P* = 0.0121) in control cells (Fig. 6*B*). CRH had no significant effect on BF in E4031‐treated cells (from 0.02 ± 0.01 to 0.10 ± 0.16, *n* = 9, *P* = 0.3790). In contrast while CRH significantly increased spike frequency in all E4031‐treated cells (from 0.37 ± 0.46 to 3.43 ± 2.80 Hz, *n* = 9, *P* = 0.0083), there was no significant effect of CRH on spike frequency in control cells (*P* = 0.6839), with only 4 of 7 control cells exhibiting any increase (Fig. 6*C*), most likely as CRH predominantly results in a transition to bursting. CRH had no effect on spike duration in control or E4031‐treated cells (Fig. 6*D*).

Although CRH increased burst frequency in all seven control and 8 of 9 E4031‐treated cells, in neither case did this reach statistical significance (*P* = 0.0778 and 0.3122 for control and E4031, respectively, Fig. 6*E*). Although CRH induced a significant increase in burst duration (from 206.95 ± 116.82 to 343.5 ± 198.46 ms, *n* = 6, *P* = 0.0181) in control cells (Fig. 6*F*), there was no significant CRH‐induced increase in burst duration in E4031‐treated corticotrophs (burst duration after CRH was 187.71 ± 87.12 ms, *n* = 8, *P* = 0.1581).

Taken together these data support the observations with CRH+AVP that the transition from spontaneous spiking to CRH‐induced bursting is blunted when Erg‐like currents are pharmacologically inhibited but that CRH can still depolarise corticotrophs resulting in an increase in spike frequency. This suggests that Erg currents normally support CRH‐induced bursting in anterior pituitary corticotrophs.

### Mathematical modelling supports a role for Erg‐like currents in supporting bursting

**Figure 7:**
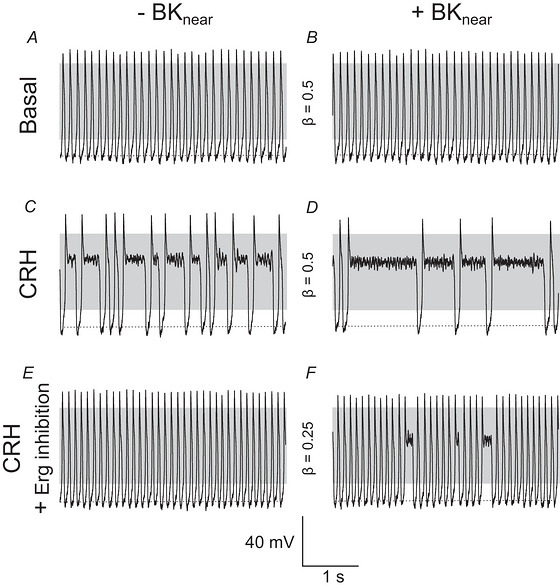
Modelling reveals Erg supports electrical bursting in corticotrophs Computer simulations of a corticotroph model that includes Erg‐like current. *A* and *B*, the model cell spikes spontaneously but does not burst under basal conditions. This is true in the absence or presence of current from BK_near_ channels. *C*, when the application of CRH (corticotrophin releasing hormone) is simulated, the model cell produces bursting even in the absence of BK_near_ channels. *D*, when BK_near_ channels are present the burst events are longer. *E*, in the absence of BK_near_ channels, a replacement of 50% of the Erg conductance with delayed rectifier conductance (β lowered from 0.5 to 0.25) eliminates CRH‐induced bursting. *F*, the BK_near_ conductance that increased the burst duration in panel *D* is insufficient to rescue bursting when the Erg conductance is reduced (β=0.25). Dotted line indicates resting membrane potential. Grey shading indicates membrane potential between −50 and +10 mV.

How might inhibition of an Erg‐like current result in suppression of CRH‐induced bursting? A requirement for an active K^+^ current to support bursting may initially appear paradoxical, especially as in other pituitary cells pharmacological inhibition of Erg‐like currents promotes membrane depolarisation and increased spike frequency. Bursting in corticotrophs and other pituitary cells relies on BK channel activity that limits the activation of other delayed rectifier potassium conductances that would normally promote repolarisation (Duncan et al., 2015; Duncan et al., 2022; Tabak et al., 2011; Van Goor et al., 2001). Here Erg‐like currents would appear to potentially play a similar role as pharmacological inhibition reduced CRH‐induced bursting. To test this idea we modified our previous mathematical model of murine corticotrophs by introducing an Erg‐like conductance with slow activation and rapid inactivation kinetics and modifying model parameters so that spontaneous action potential spiking and CRH‐induced bursting are supported (see Methods). In this model under basal conditions the cell produces spiking but no bursting. This is true whether current from BK channels located near Ca^2+^ channels (BK_near_) is excluded (Fig. 7*A*) or included (Fig. 7*B*).

When application of CRH alone is simulated, the model cell produces pseudoplateau bursting. This is true even without BK_near_ channels (Fig. 7*C*), demonstrating that the Erg‐like current can play a role similar to that of BK channels in the production of bursting. When conductance from BK_near_ channels is included, bursts are increased in duration (Fig. 7*D*), demonstrating that BK_near_ plays a role in burst production whether Erg‐like current is present in the cell or not. Due to the heterogeneity in the level of Erg current that we reported in Fig. 2*C*, the fact that both Erg and BK current promote bursting adds a level of redundancy to the CRH response.

Partial reduction in Erg‐like current is simulated in the model by reducing the parameter β from 0.5 to 0.25. This reduces the Erg conductance by one half and replaces it with delayed rectifier conductance. Under these conditions the bursting pattern is replaced by spiking. This is true when BK_near_ channels are absent (Fig. 7*E*) and even when they are present (Fig. 7*F*), although in this latter case a few bursting events occur. Overall, then, the simulations indicate that Erg is important for burst production, complementing the effect of BK_near_ channels.

## Discussion

The mRNA encoding Erg1 channels (*Kcnh2*) is enriched in the anterior pituitary (Sanchez‐Conde et al., 2022), and Erg‐like currents have been reported to play an inhibitory role in both lactotrophs and gonadotrophs by limiting membrane depolarisation and increases in action potential spiking (Bauer, 1998; Hirdes et al., 2010; Storey et al, 2002). However their role in pituitary cells that exhibit robust bursting is not known. Here we show that both male and female murine corticotrophs functionally express E4031‐sensitive currents, with biophysical characteristics of the Erg subfamily of EAG channels (Bauer & Schwarz, 2018; Sanchez‐Conde et al., 2022), that do not control membrane depolarisation or action potential spiking. Although these Erg currents are not regulated by CRH, or AVP, Erg currents support CRH‐induced bursting in corticotrophs. This reveals the important context dependence of Erg channel function in excitable endocrine cells and has important implications for the interaction of the stress axis with cardiovascular function.

### Erg‐like currents support CRH‐induced bursting in murine corticotrophs

As *Kcnh2* was the predominant Erg mRNA expressed in murine corticotrophs along with the mRNA (*Kcne2*) encoding the Erg accessory subunit MiRP1, it is likely that E4031‐sensitive currents are dominated by Erg1. However as lower levels of *Erg2* (*Kcnh6*) and *Erg3* (*Kcnh7*) mRNA were also expressed, and Erg channels can heteromultimerise and are sensitive to E4031, we cannot eliminate that other Erg conductances also play an important role. Indeed considerable variability in both E4031‐sensitive current density and current kinetics was observed between individual corticotrophs. In particular both male and female corticotrophs exhibit Erg currents with either fast or slow deactivation. Multiple different mechanisms may underlie the observed differences in Erg current kinetics between cells. Firstly different alternate transcripts, due to different start sites in the promoter, or alternative mRNA splicing of *Kcnh2* can significantly modify deactivation kinetics, with variants disrupting the N‐terminal Per‐Arnt‐Sim (PAS) and C‐terminal cyclic nucleotide‐binding homology (CNBH) domain interactions exhibiting much faster activation and deactivation without affecting *V*_50max_ (Sanchez‐Conde et al., 2022; Codding & Trudeau, 2019; Wang et al, 1998). In addition co‐expression with Kcne2 (Mirp1) can both reduce Kcnh2 single‐channel conductance and accelerate deactivation (Abbott et al, 1999). Alternatively although our RNA‐seq analysis reveals that *Kcnh2 *mRNA is the dominant transcript across the corticotroph *population* in both males and females, we cannot exclude that Kcnh or Kcne subunits are differentially expressed at a *single‐cell* level. For example *Kcnh*7 mRNA is expressed at low levels in the population, and Kcnh7 homomultimers have much faster deactivation kinetics than Kcnh2 homomultimers (Sanchez‐Conde et al., 2022; Wimmers et al., 2002). Clearly these are important questions to be addressed in the future together with understanding the functional role of the relatively high expression of *Elk1* (*Kcnh8*) mRNA in corticotrophs that encodes non‐inactivating potassium channels.

Surprisingly inhibition of Erg‐like currents with either E4031 or DOF (that also inhibits some Eag channels) had no significant effect on resting membrane potential or spontaneous action potential behaviour in corticotrophs. This contrasts with that observed in lactotrophs and gonadotrophs as well as some other endocrine cells such as chromaffin cells (Bauer, 1998; Bauer et al., 1999; Gullo et al., 2003; Hardy et al., 2009; Hirdes et al., 2010; Kirchberger et al., 2006; Lecchi et al., 2002; Rosati et al., 2000; Schafer et al., 1999; Storey et al., 2002, 2006). Erg inhibition also had no significant effect on membrane depolarisation induced by CRH+AVP or CRH alone, suggesting Erg does not play an important role in the early phase of membrane depolarisation by these secretagogues and in agreement with the lack of effect of E4031 or DOF on resting membrane potential.

However although CRH+AVP had no effect on Erg‐like current density or *V*_50max_ in corticotrophs, pharmacological inhibition of Erg suppressed both CRH+AVP and CRH‐induced bursting. This apparently paradoxical role of a voltage‐gated potassium channel in supporting bursting behaviour in anterior pituitary cells is reminiscent of the promotion of bursting by large‐conductance calcium‐ and voltage‐activated (BK) channels in a variety of pituitary cell types, including corticotrophs (Duncan et al., 2015; Tabak et al., 2011; Van Goor et al., 2001). In these systems a pool of BK channels (BK_near_) localised close to voltage‐gated calcium channels are critical for supporting bursting by limiting membrane depolarisation to reduce activation of other voltage‐gated potassium channels that would act to depolarise the cell. In corticotrophs our studies suggest that Erg‐like currents play a complimentary role. Indeed using mathematical modelling CRH‐induced bursting can be supported in the absence of BK_near_ in the presence of an Erg‐like conductance. Important in this regard is the interplay with the activation kinetics of potassium conductances during the initiation of a burst to allow the membrane potential to oscillate at more depolarised potentials creating a long‐duration burst. BK_near_ provides a fast‐activating potassium conductance to limit depolarisation. In contrast as Erg channels activate slowly but inactivate rapidly, cells with a significant Erg current would have a smaller repolarising current to facilitate burst initiation. Furthermore pharmacological inhibition of Erg‐like currents resulted in a reduction in CRH‐induced burst duration, suggesting Erg may play a role in both the initiation and maintenance of CRH‐induced bursts.

Erg currents have also been proposed to play a role in a different mode of burst‐plateau electrical bursting in rat and mouse subthalamic neurones (STNs) (Huang et al, 2017). This bursting is characterised by large‐amplitude, high‐frequency action potentials that accommodate during a period of depolarisation, with increased bursting frequency characteristic of STN behaviour in models of Parkinson's disease. In these neurones inhibition of Erg with submaximal doses of E4031 can both reduce spontaneous burst rates and convert burst discharges into tonic action potentials (Huang et al, 2017). Taken together with our data in corticotrophs, these data emphasise the importance of context dependence in the functional role of Erg channels in controlling excitability in both endocrine cells and central neurones.

### Potential implications for hypothalamic‐pituitary‐adrenal‐axis cardiovascular interactions

The role of Erg channels in controlling anterior pituitary corticotroph physiology has potential implications for both the control of the stress axis and cardiovascular function. The classical role of ERG1 (KCNH2) channels is to control the duration of the cardiac ventricular action potential. Indeed patients with loss‐of‐function mutations in the *KCNH2* gene exhibit elongation of the cardiac action potential (long QT syndrome (LQTS), LQT2). The final output of the hypothalamic‐pituitary‐adrenal (HPA) axis, the circulating glucocorticoids, is also implicated in reducing the duration of the cardiac action potential and thus potentially counteracting the effects of loss‐of‐function *KCNH2 m*utations. For example it has been known for many years that patients with adrenal insufficiency that may be primary as a result of Addison's disease (Lang et al., 2012; Somerville et al., 1951) or secondary to adrenocorticotrophin hormone (ACTH) deficiency (Chakraborty et al., 2022; Oshizaka et al., 2025; Suzuki & Hayashi, 2016) have elongated cardiac action potential duration (LQTS). The elongation of the cardiac action potential in part may be corrected by glucocorticoid replacement (Bandorski et al., 2023; Hartog & Joplin, 1968; Somerville, 1950). Glucocorticoids can regulate Erg1 biosynthesis and function (Lamothe & Zhang, 2013); however whether such a mechanism mediates the effect of glucocorticoids on the cardiac action potential or may control Erg expression in corticotrophs remains to be determined. Irrespective of this if Erg channel dysregulation in anterior pituitary corticotrophs results in suppression of glucocorticoid output, as would be predicted by limiting CRH‐induced bursting in corticotrophs, this reduction in circulating glucocorticoids may further exacerbate LQTS in patients with *KCNH2 m*utations. However studies investigating circulating glucocorticoid levels in patients with LQT2 or other LQTS have, to the best of our knowledge, not been systematically examined.

In summary we reveal a novel role for Erg‐like channels in controlling CRH‐induced bursting in murine anterior pituitary corticotrophs. Understanding the role of Erg in the stress axis and the interplay between the HPA axis and cardiovascular function in health and disease is warranted.

## Additional information

## Competing interests

All authors declare that they have no competing interests/conflicts of interest in this work.

## Author contributions

S.V.N., P.J.D. and N.R. contributed to the acquisition, analysis and interpretation of data for the work. S.V.N., P.J.D., N.R., P.L.T., R.B. and M.J.S. contributed to the conception, design, interpretation and drafting of the work and revised it critically for important intellectual content. All authors have approved the final version of the manuscript and agree to be accountable for all aspects of the work in ensuring that questions related to the accuracy or integrity of any part of the work are appropriately investigated and resolved; all persons designated as authors qualify for authorship, and all those who qualify for authorship are listed.

## Funding

The work was supported by MRC grants MR/V012290/1 and MR/R010668 to P.J.D., N.R., P.L.T and M.J.S. S.V.N. was in part supported by an NS‐ICAR International Fellowship, BSN COVID‐19 exceptional grant and Edinburgh Global Research scholarship (EGRS).

## Supporting information


Peer Review History

## Data Availability

Data supporting the results presented in this article are included in the figures. Computer codes for model are available at www.math.fsu.edu/~bertram/software/pituitary and https://github.com/rbertram.
