# Supplementary material for: Erg currents support electrical bursting in murine anterior pituitary corticotrophs
Source: J Physiol. 2026 May 11;604(12):4834–50. doi: 10.1113/JP290793 (PMC13267669; doi:10.1113/JP290793)
Supplement: Supplementary file 1 — Peer Review History [file TJP-604-4834-s001.pdf]

The Journal of Physiology

Erg currents support electrical bursting in murine anterior pituitary corticotrophs

Sooraj V Nair, Nicola Romanò, Peter J Duncan, Paul Le Tissier, Richard Bertram, and Michael J Shipston
DOI: 10.1113/JP290793

Corresponding author(s): Michael Shipston (mike.shipston@ed.ac.uk)

The following individual(s) involved in review of this submission have agreed to reveal their identity: Jürgen R Schwarz (Referee #2)

Review Timeline:

Submission Date:	21-Dec-2025
Editorial Decision:	22-Jan-2026
Revision Received:	14-Mar-2026
Editorial Decision:	08-Apr-2026
Revision Received:	13-Apr-2026
Accepted:	15-Apr-2026

Senior Editor: Peying Fong

Reviewing Editor: Theodore Cummins

Transaction Report:

(Note: With the exception of the correction of typographical or spelling errors that could be a source of ambiguity, letters and reports are not edited. Depending on transfer agreements, referee reports obtained elsewhere may or may not be included in this compilation. Referee reports are anonymous unless the Referee chooses to sign their reports.)

Re: JP-RP-2025-290793 "**Erg currents support electrical bursting in murine anterior pituitary corticotrophs**" by Sooraj V Nair, Nicola Romanò, Peter J Duncan, Paul Le Tissier, Richard Bertram, and Michael J Shipston

Dear Dr Shipston,

Thank you for submitting your manuscript to The Journal of Physiology. It has been assessed by a Reviewing Editor and by 2 expert referees and we are pleased to tell you that it is potentially acceptable for publication following satisfactory major revision.

Please advise your co-authors of this decision as soon as possible.

The referee reports are copied at the end of this email.

Please address all the points raised and incorporate all requested revisions or explain in your Response to Referees why a change has not been made. We hope you will find the comments helpful and that you will be able to return your revised manuscript within 2 months. If your article is NOT for a Special Issue, you may have 9 months to revise. If you require an extension, please contact journal staff: jp@physoc.org. Please note that this letter does not constitute a guarantee for acceptance of your revised manuscript.

Your revised manuscript should be submitted online using the link in your Author Tasks: Link Not Available. This link is accessible via your account as Corresponding Author; it is not available to your co-authors. If this presents a problem, please contact journal staff (jp@physoc.org). Image files from the previous version are retained on the system. Please ensure you replace or remove any files that are being revised.

If you do not wish to submit a revised version of your manuscript, you must inform our journal staff (jp@physoc.org) or reply to this email to request withdrawal. Please note that a manuscript must be formally withdrawn from the peer review process at one journal before it may be submitted to another journal.

TRANSPARENT PEER REVIEW POLICY: To improve the transparency of its peer review process, The Journal of Physiology publishes online as supporting information the peer review history of all articles accepted for publication. Readers will have access to decision letters, including Editors' comments and referee reports, for each version of the manuscript, as well as any author responses to peer review comments. Referees can decide whether or not they wish to be named on the peer review history document.

ABSTRACT FIGURES: Authors are expected to use The Journal's premium BioRender account to create/redraw their Abstract Figures. Information on how to access this account is here: https://physoc.onlinelibrary.wiley.com/journal/14697793/biorender-access.

This will enable Authors to create and download high-resolution figures. If authors have used the free BioRender service, they can use the instructions provided in the link above to download a high-resolution version suitable for publication.

The link provided should only be used for the purposes of this submission. Authors will be charged for figures created on this account if they are not related to this manuscript submission.

LANGUAGE EDITING AND SUPPORT FOR PUBLICATION: If you would like help with English language editing, or other article preparation support, Wiley Editing Services offers expert help, including English Language Editing, as well as translation, manuscript formatting, and figure formatting at www.wileyauthors.com/eeo/preparation. You can also find resources for Preparing Your Article for general guidance about writing and preparing your manuscript at www.wileyauthors.com/eeo/prepresources.

RIGOUR AND REPRODUCIBILITY: We recommend authors consult The Journal's Rigour and Reproducibility requirements to ensure that their manuscript is suitable for publication: https://physoc.onlinelibrary.wiley.com/pb-assets/hub-assets/physoc/documents/TJP-Rigour-and-Reproducibility-Requirements.pdf

REVISION CHECKLIST:

Check that your Methods section conforms to journal policy: https://jp.msubmit.net/cgi-bin/main.plex?form_type=display_requirements#methods.

Check that data presented conforms to the statistics policy: https://jp.msubmit.net/cgi-bin/main.plex?form_type=display_requirements#statistics.

Upload a full Response to Referees file. To create your 'Response to Referees': copy all the reports, including any comments from the Senior and Reviewing Editors, into a Microsoft Word, or similar, file and respond to each point, using font or background colour to distinguish comments and responses and upload as the required file type.

Please upload two versions of your manuscript text: one with all relevant changes highlighted and one clean version with no changes tracked. The manuscript file should include all tables and figure legends, but each figure/graph should be uploaded as separate, high-resolution files.

You may also upload:

- 'Potential Cover Art' for consideration as the issue's cover image
- Appropriate Supporting Information (Video, audio or data set: see https://jp.msubmit.net/cgi-bin/main.plex?form_type=display_requirements#supp).

We look forward to receiving your revised submission.

If you have any queries, please reply to this email and we will be pleased to advise.

Yours sincerely,

Peying Fong
Senior Editor
The Journal of Physiology

EDITOR COMMENTS

Reviewing Editor:

Ethics Concerns:
The Methods section must begin with the subheading "Ethical Approval". Key information on the animal approvals and methods should be in this section at the beginning of the methods section.

Comments for Authors to ensure the paper complies with the Statistics Policy:
The reviewers have identified key issues relating to the graphs and data presentation that need to be rectified.

Comments to the Author:
This study could potentially have significant physiological importance. The data suggest a role for Erg currents in supporting electrical bursting in corticotrophs by induced CRH. The data are supportive, but multiple issues raised by both reviewers, largely in the presentation, presentation and interpretation of the data, need to be addressed before further consideration of the manuscript. These revisions are feasible and would enhance the impact of the study.

Senior Editor:

Comments to the Author:
Review of your manuscript "Erg currents support electrical bursting in murine anterior pituitary corticotrophs" is now complete. Attached are detailed critiques of two expert referees, as well as the Reviewing Editor's summary. Although all concur on its potential for impact, the attached critiques highlight the need for tighter presentation and deeper analysis of the reported findings. Taken together, both referees offer actionable suggestions for improving both methodological and analytical rigor, as well as refining clarity of presentation. Please use these comments to guide you as you prepare your revised manuscript.

We look forward to receiving your revision and thank you for your contribution to The Journal of Physiology.

REFEREE COMMENTS

Referee #1:

This manuscript presents novel and compelling evidence that ERG-like currents support CRH-induced bursting in murine anterior pituitary corticotrophs. The combination of electrophysiology, pharmacology, and mathematical modeling is a major strength. The work fills a gap in the field, given that ERG currents have been previously characterized in lactotrophs and GH3 cell lines but not in identified corticotrophs.

I recommend including the recent review by Tagliavini et al. (Front Mol Neurosci, 2022; 15:890368), which provides an up to date and comprehensive overview of ERG1 channel expression and function, including explicit documentation of KCNH2

expression within the pituitary gland. This article contextualizes the current findings within broader ERG physiology and would strengthen both the Introduction and Discussion sections.

Regarding methods and design:

1. While V_{50} values derived from the activation curves were reported, whether or not a Boltzmann function was used and the slope factor (k) were not indicated. Because the slope provides essential information about voltage sensitivity and channel gating, and is a standard parameter when reporting activation curves, the fitting model and both V_{50} and slope values should be specified in the results/ figure legend. This is important for reproducibility and for comparison with prior ERG biophysical studies.

2. The manuscript mixes within-cell (paired) comparisons (baseline → CRH or CRH+AVP) with between-group (unpaired) comparisons (control vs E4031 vs DOF). While this is a perfectly acceptable design, the distinction is not stated explicitly and can be misread-especially because representative traces look sequential and several sentences use phrasing that sounds like the same cells were used. Paired tests should be used if same cell comparisons are made.

As for experimental data:

1. n-value inconsistencies appear. For example, there is a discrepancy between the n values for females reported in the figure 3 legend and those shown in the Figure 3 box plot.

Also, in fig 3 the normalized peak currents reported for male cells are "n = 4 and 6 for control and CRH + AVP treated cells respectively". but the box plot in C showed n=6 and 6 for male control and crh + avp. why Were there less number of cells used for mean peak current?

2. ERG currents start activating near -50 mV but the IV shows activation at -80 and -70.

Minor suggestions:

1.

There are quite a few repetitions in the manuscript:

For example:

Page 3: "pharmacological inhibition of Erg channels can promote basal and/or secretagogue-evoked cell depolarisation and an increase in action potential firing in lactotrophs and gonadotrophs"

And a few lines later: "Pharmacological inhibitors such as the type III antiarrhythmic methanesulfonanilide E4031 that inhibits all Erg, but not Eag currents (Shi et al., 1997; Herzberg et al., 1998), has revealed an important role for Erg-like currents in the control of both native lactotroph and gonadotroph electrical excitability"

page 18 "Here we show that both male and female murine corticotrophs functionally express E4031-sensitive Erg-like currents " and "Both male and female murine corticotrophs expressed functional E4031-sensitive currents"

The manuscript reports resting membrane potential values for control and E4031-treated cells in separate sections (spontaneous activity and CRH+AVP experiments and CRH alone), this appears repetitive.

2. Please check for missing commas throughout the document: For example:

- Page 14: "Under these conditions in control cells"

- Page 4: "In murine corticotrophs spontaneous"

- "In 7/7 control cells CRH induced a small depolarisation of the"

- "In contrast as Erg channels activate slowly but rapidly inactivate,"

3. "involved in CRH+AVP induce -> induced

4. "has revealed" → "have revealed" end of page 3

5. Gene names should always be italicized eg Kcnh2 page 20

6. G-protein coupled receptors abbreviation should be included when it first appears page 4

7. Check whole document for appropriate and consistent capitalization example "erg" page 4

8. Missing references: "GPCR-mediated inhibition of Erg currents is typically characterised by a decrease in maximum current amplitude as well as a right-shift in the V50max to more depolarised potentials." Page 12 and

"In other endocrine cells, including anterior pituitary cells, pharmacological inhibition of Erg-like currents has been reported to depolarise membrane potential and enhance spontaneous action potential frequency. " page 13

Referee #2:

This paper describes the properties of erg K currents in male and female mouse anterior pituitary corticotroph cells. In the first place the authors show that in male and female corticotrophs mRNA for Kcnh2 is present, whereas mRNA of Kcnh6 and Kcnh7 is almost absent. Therefore, corticotroph erg currents are likely mediated by erg1 K channels. The authors then performed electrophysiology and show that erg currents of corticotrophs are very variable, e.g. erg current densities and current kinetics varied a lot. Astonishingly, inhibition of corticotroph erg currents by E4031, a pharmacological blocker well known for its selective blocking of all three erg channels, did not influence resting potential and spontaneous firing of action potentials. The main finding of the paper is that CRH induces bursting in the presence of erg currents, whereas erg current inhibition by E4031 stopped bursting and irregular tonic spiking occurred. This is an important finding. Since bursting in corticotrophs is also initiated and maintained by Ca2+- and voltage-activated BK channels, this new finding shows that bursting is supported by activation of two different types of ion channels.

Analysis of ion channels responsible for the initiation of bursting and tonic firing and the transition between both is high physiological importance. A similar function of erg currents to induce bursting has recently been observed in dopaminergic subthalamic neurons (Huang et al., Sci. Adv. 2017;3: e1602272). Anyhow, this paper is unsatisfactory for several reasons:

1. The biophysical properties of erg currents recorded from male and female corticotrophs exhibit large differences. These differences are neither explained nor analyzed. 2. Since both, by which both channel types contribute to bursting are not further analyzed and discussed. The mathematical model described in the paper does not provide further analysis, especially it does not show any bursting and accommodation.

The authors should consider the following points:

1. Figure 1 and page 10, line 5: The Kcne mRNA level of female corticotrophs is larger than that of male corticotrophs. Is it significantly larger? The possible effect of MIRP1 on erg currents should be discussed.

2. Page 11: what is the meaning of "the proportion of peak biophysically isolated current" ?

3. Figure 2A, B illustrates the differences between erg currents recorded from male and female corticotrophs. Recorded at the same membrane potentials, the kinetics of male erg currents are much faster than female erg currents. At least, at the mRNA level there seems to be no difference. Presumably, at the protein level there is also no difference. The activation curves of male and female erg currents are also very similar. Nevertheless, the shape of the activation curves suggests that

two different activation curves may exist, superimposed on each other. The authors should try to fit the activation data by two sigmoidal curves. In addition, the authors should evaluate the voltage dependence of the time constants. It will be very unsatisfactory, if there is no explanation for these strong differences. One solution to this problem could be to concentrate just on male or female corticotrophs.

4. Page 12, lines 9 and 12: references should be added to statements taken from the literature. This is true for several other statements throughout the text which also need to be verified by references.

5. Figure 3C: Although there was no change in V50max determined in control cells, was there any change in the shape of the activation curves of erg currents in the presence of CRH and AVP?

6. Figure 4: The letters are missing to identify the six panels.

A, B: At least the recordings selected to represent basal conditions show a small depolarization in the recording of the E-4031-treated corticotroph. In addition, the number of spikes doubled in the presence of E4031. Obviously, these recordings are not representative. On the other hand, such a depolarization is plausible, since the activation curves of Fig. 2D show a steady state erg current at the resting potential of -45 mV (female) and -50 mV (male).

C-F: These data are very nice and convincing. I suggest to indicate the level of the resting potential in all recordings. This would help to estimate the absolute value of the resting potential and the magnitude of the transient hyperpolarizations following the bursts.

7. Page 13: The resting potential of female corticotrophs is about 5 mV more depolarized than that of male corticotrophs. Is there any explanation for this difference?

8. Figure 5 and 6: Both figures are difficult to understand. One improvement could be to add the levels of statistical significance to the figures themselves. Another suggestion is to remove the dofetilide data (just for clarity), and to summarize them in the text.

9. What is the rationale for studying dofetilide?

10. Figure 7: The labeling of the panels (A-F) as well as the time and potential axes are missing. As in Figure 4, it would be helpful to indicate the level of the resting potential for each calculation.

C, D: In addition to the level of the resting potential the level of the plateau potentials should be indicated. Can the model generate bursts and accommodation during bursts?

END OF COMMENTS

JP-RP-2025-290793
Title: Erg currents support electrical bursting in murine anterior pituitary corticotrophs
by Nair et al.

This paper describes the properties of erg K currents in male and female mouse anterior pituitary corticotroph cells. In the first place the authors show that in male and female corticotrophs mRNA for *Kcnh2* is present, whereas mRNA of *Kcnh6* and *Kcnh7* is almost absent. Therefore, corticotroph erg currents are likely mediated by erg1 K channels. The authors then performed electrophysiology and show that erg currents of corticotrophs are very variable, e.g. erg current densities and current kinetics varied a lot. Astonishingly, inhibition of corticotroph erg currents by E4031, a pharmacological blocker well known for its selective blocking of all three erg channels, did not influence resting potential and spontaneous firing of action potentials. The main finding of the paper is that CRH induces bursting in the presence of erg currents, whereas erg current inhibition by E4031 stopped bursting and irregular tonic spiking occurred. This is an important finding. Since bursting in corticotrophs is also initiated and maintained by Ca^{2+}- and voltage-activated BK channels, this new finding shows that bursting is supported by activation of two different types of ion channels.

Analysis of ion channels responsible for the initiation of bursting and tonic firing and the transition between both is high physiological importance. A similar function of erg currents to induce bursting has recently been observed in dopaminergic subthalamic neurons (Huang et al., Sci. Adv. 2017;3: e1602272). Anyhow, this paper is unsatisfactory for several reasons: 1. The biophysical properties of erg currents recorded from male and female corticotrophs exhibit large differences. These differences are neither explained nor analyzed. 2. Since both, BK channels and erg K channels, are involved in bursting, the different ionic mechanisms by which both channel types contribute to bursting are not further analyzed and discussed. The mathematical model described in the paper does not provide further analysis, especially it does not show any bursting and accommodation.

The authors should consider the following points:

1. Figure 1 and page 10, line 5: The Kcne mRNA level of female corticotrophs is larger than that of male corticotrophs. Is it significantly larger? The possible effect of MIRP1 on erg currents should be discussed.

2. Page 11: what is the meaning of "the proportion of peak biophysically isolated current" ?

3. Figure 2A, B illustrates the differences between erg currents recorded from male and female corticotrophs. Recorded at the same membrane potentials, the kinetics of male erg currents are much faster than female erg currents. At least, at the mRNA level there seems to be no difference. Presumably, at the protein level there is also no difference. The activation curves of male and female erg currents are also very similar. Nevertheless, the shape of the activation curves suggests that two different activation curves may exist, superimposed on each other. The authors should try to fit the activation data by two sigmoidal curves. In addition, the authors should evaluate the voltage dependence of the time constants. It will be very unsatisfactory, if there is no explanation for these strong differences. One solution to this problem could be to concentrate just on male or female corticotrophs.

4. Page 12, lines 9 and 12: references should be added to statements taken from the literature. This is true for several other statements throughout the text which also need to be verified by references.

5. Figure 3C: Although there was no change in V_{50max} determined in control cells, was there any change in the shape of the activation curves of erg currents in the presence of CRH and AVP?

6. Figure 4: The letters are missing to identify the six panels.
A, B: At least the recordings selected to represent basal conditions show a small depolarization in the recording of the E-4031-treated corticotroph. In addition, the number of spikes doubled in the presence of E4031. Obviously, these recordings are not representative. On the other hand, such a depolarization is plausible, since the activation curves of Fig. 2D show a steady state erg current at the resting potential of -45 mV (female) and -50 mV (male).
C-F: These data are very nice and convincing. I suggest to indicate the level of the resting potential in all recordings. This would help to estimate the absolute value of the resting potential and the magnitude of the transient hyperpolarizations following the bursts.

7. Page 13: The resting potential of female corticotrophs is about 5 mV more depolarized than that of male corticotrophs. Is there any explanation for this difference?

8. Figure 5 and 6: Both figures are difficult to understand. One improvement could be to add the levels of statistical significance to the figures themselves. Another suggestion is to remove the dofetilide data (just for clarity), and to summarize them in the text.

9. What is the rationale for studying dofetilide?

10. Figure 7: The labeling of the panels (A-F) as well as the time and potential axes are missing. As in Figure 4, it would be helpful to indicate the level of the resting potential for each calculation.
C, D: In addition to the level of the resting potential the level of the plateau potentials should be indicated. Can the model generate bursts and accommodation during bursts?

Response to Editor and referees

We would like to thank the Editors and reviewers for their very supportive and constructive comments. We have addressed all issues, including new data and revised analysis/presentation along with additional clarification of approach as well as enhanced discission of key points raised.

We believe this has improved the manuscript and hope that it is now suitable for publication in The Journal of Physiology.

Mike Shipston, on behalf of all authors

EDITOR COMMENTS

Reviewing Editor:

Ethics Concerns:
The Methods section must begin with the subheading "Ethical Approval". Key information on the animal approvals and methods should be in this section at the beginning of the methods section.

Edited as requested

Comments for Authors to ensure the paper complies with the Statistics Policy:
The reviewers have identified key issues relating to the graphs and data presentation that need to be rectified.

These have been addressed in comments below

Comments to the Author:
This study could potentially have significant physiological importance. The data suggest a role for Erg currents in supporting electrical bursting in corticotrophs by induced CRH. The data are supportive, but multiple issues raised by both reviewers, largely in the presentation, presentation and interpretation of the data, need to be addressed before further consideration of the manuscript. These revisions are feasible and would enhance the impact of the study.

Thankyou, we have addressed all concerns of reviewers including additional data analysis, interpretation and discussion

Senior Editor:

Comments to the Author:
Review of your manuscript "Erg currents support electrical bursting in murine anterior pituitary corticotrophs" is now complete. Attached are detailed critiques of two expert referees, as well as the Reviewing Editor's summary. Although all concur on its potential for impact, the attached critiques highlight the need for tighter presentation and deeper analysis of the reported findings. Taken together, both referees offer actionable suggestions for improving both methodological and analytical rigor, as well as refining clarity of presentation. Please use these comments to guide you as you prepare your revised manuscript.

Thankyou, we have addressed all concerns of reviewers including additional data analysis, interpretation and discussion

----

REFEREE COMMENTS

Referee #1:

This manuscript presents novel and compelling evidence that ERG-like currents support CRH-induced bursting in murine anterior pituitary corticotrophs. The combination of electrophysiology, pharmacology, and mathematical modeling is a major strength. The work fills a gap in the field, given that ERG currents have been previously characterized in lactotrophs and GH3 cell lines but not in identified corticotrophs.

Thank you for your supportive comments

I recommend including the recent review by Tagliavini et al. (Front Mol Neurosci, 2022; 15:890368), which provides an up to date and comprehensive overview of ERG1 channel expression and function, including explicit documentation of KCNH2 expression within the pituitary gland. This article contextualizes the current findings within broader ERG physiology and would strengthen both the Introduction and Discussion sections.

We believe the referee is referring to the excellent review by Sanchez-Conde FG, Jimenez-Vazquez EN, Auerbach DS & Jones DK. (2022). The ERG1 K(+) Channel and Its Role in Neuronal Health and Disease. *Front Mol Neurosci* **15,** 890368. This review was included in the original manuscript throughout including on page 2 of introduction to highlight physiological roles as well as supporting expression of Kcnh2 in the pituitary.

Regarding methods and design:

1. While V_{50} values derived from the activation curves were reported, whether or not a Boltzmann function was used and the slope factor (k) were not indicated. Because the slope provides essential information about voltage sensitivity and channel gating, and is a standard parameter when reporting activation curves, the fitting model and both V_{50} and slope values should be specified in the results/ figure legend. This is important for reproducibility and for comparison with prior ERG biophysical studies.

We have now included in the methods section that the activation curve data were best fitted by a Boltzmann fit to determine V50 and slope. We have now included the slope factor (k) for Erg current recordings from male and female corticotrophs in the corresponding results text. For males the slope for E4031 sensitive currents was 7.93 +_ 0.35 (n = 9) and for females 8.49 +_2.29 (n= 4), the slope was not significantly different between the sexes.

2. The manuscript mixes within-cell (paired) comparisons (baseline → CRH or CRH+AVP) with between-group (unpaired) comparisons (control vs E4031 vs DOF). While this is a perfectly acceptable design, the distinction is not stated explicitly and can be misread-especially because representative traces look sequential and several sentences use phrasing that sounds like the same cells were used. Paired tests should be used if same cell comparisons are made.

Thank you for raising this important point. We have clarified throughout including in the methods section

As for experimental data:

1. n-value inconsistencies appear. For example, there is a discrepancy between the n values for females reported in the figure 3 legend and those shown in the Figure 3 box plot.

Thank you for your careful analysis revealing this error in n number. In panel B of Figure 3 we used the single pulse protocol to determine the time course of effect of CRH + AVP on currents in female corticotrophs, this was analysed in 5 cells with control perfusion and 7 cells with CRH+AVP. In panel C we used the separate multistep potential protocol to determine deltaV50max under each condition and in this case this was performed in n = 3 cells for control and n = 5 for cells treated with CRH+AVP. We have modified the figure legend to correct and clarify this.

Also, in fig 3 the normalized peak currents reported for male cells are "n = 4 and 6 for control and CRH + AVP treated cells respectively". but the box plot in C showed n=6 and 6 for male control and crh + avp. why Were there less number of cells used for mean peak current?

In panel A of Figure 3 we used the single pulse protocol to determine the time course of effect of CRH + AVP on currents, this was analysed in 4 cells with control perfusion and 6 cells with CRH+AVP. In panel C we used the multistep potential protocol to determine deltaV50max under each condition and in this case n = 6 for both control and for cells treated with CRH+AVP. We have modified the figure legend to clarify.

2. ERG currents start activating near -50 mV but the IV shows activation at -80 and -70.

ERG1 currents typically activate between -60 to -50 mV however ERG3 can activate at lower potentials. While in the majority of cases currents activate between -60 mV and -50 mV, in a small number of our recordings we do see measurable current at -70 mV under the high potassium conditions used (Sturm et al (2005) JPhysiol 564: 329-345), reflected in the greater variation of the current at these potentials in the combined current-voltage plot. Alternatively, it could reflect contribution from other conductances such as via Kcnh8 channels that activate at much more negative potentials. Whether this is due to the presence of ERG3 current, or other conductances, cannot be determined in our assays.

Minor suggestions:

1.

There are quite a few repetitions in the manuscript:

For example:

Page 3: "pharmacological inhibition of Erg channels can promote basal and/or secretagogue-evoked cell depolarisation and an increase in action potential firing in lactotrophs and gonadotrophs"
Thank you for the suggestion we have edited the text to make more concise

And a few lines later: "Pharmacological inhibitors such as the type III antiarrhythmic methanesulfonanilide E4031 that inhibits all Erg, but not Eag currents (Shi et al., 1997; Herzberg et al., 1998), has revealed an important role for Erg-like currents in the control of both native lactotroph and gonadotroph electrical excitability"
Thank you for the suggestion, we have edited the text to make more concise

page 18 "Here we show that both male and female murine corticotrophs functionally express E4031-sensitive Erg-like currents " and "Both male and female murine corticotrophs

expressed functional E4031-sensitive currents"

Thank you for the suggestion, we have edited the text to make more concise

The manuscript reports resting membrane potential values for control and E4031-treated cells in separate sections (spontaneous activity and CRH+AVP experiments and CRH alone), this appears repetitive.

As these experiments were performed differently (for effects of E4031 on spontaneous activity membrane potential was determined before and after E4031 in the same cell whereas for experiments with CRH + AVP cells were pretreated with E4031 or vehicle control in different cells before application of CRH+AVP in the same cell) we feel it is important to report the membrane potentials under the corresponding conditions

2. Please check for missing commas throughout the document: For example:

- Page 14: "Under these conditions in control cells"
Thank you – this is now edited

- Page 4: "In murine corticotrophs spontaneous"
Thank you – this is now corrected

- "In 7/7 control cells CRH induced a small depolarisation of the"
Thank you – this is now corrected

- "In contrast as Erg channels activate slowly but rapidly inactivate,"
Thank you – this is now corrected

3. "involved in CRH+AVP induce -> induced
Thank you – this is now corrected

4. "has revealed" → "have revealed" end of page 3
Thank you – this is now corrected

5. Gene names should always be italicized eg Kcnh2 page 20
Thank you – this is now corrected and checked throughout

6. G-protein coupled receptors abbreviation should be included when it first appears page 4
Thank you – this is now corrected

7. Check whole document for appropriate and consistent capitalization example "erg" page 4
Thank you – this is now corrected and checked throughout

8. Missing references: "GPCR-mediated inhibition of Erg currents is typically characterised by a decrease in maximum current amplitude as well as a right-shift in the V50max to more depolarised potentials." Page 12 and
Thank you - We have now included supporting references

"In other endocrine cells, including anterior pituitary cells, pharmacological inhibition of Erg-like currents has been reported to depolarise membrane potential and enhance spontaneous action potential frequency. " page 13

Thank you - We have now included supporting references

Referee #2:

This paper describes the properties of erg K currents in male and female mouse anterior pituitary corticotroph cells. In the first place the authors show that in male and female corticotrophs mRNA for Kcnh2 is present, whereas mRNA of Kcnh6 and Kcnh7 is almost absent. Therefore, corticotroph erg currents are likely mediated by erg1 K channels. The authors then performed electrophysiology and show that erg currents of corticotrophs are very variable, e.g. erg current densities and current kinetics varied a lot. Astonishingly, inhibition of corticotroph erg currents by E4031, a pharmacological blocker well known for its selective blocking of all three erg channels, did not influence resting potential and spontaneous firing of action potentials. The main finding of the paper is that CRH induces bursting in the presence of erg currents, whereas erg current inhibition by E4031 stopped bursting and irregular tonic spiking occurred. This is an important finding. Since bursting in corticotrophs is also initiated and maintained by Ca2+- and voltage-activated BK channels, this new finding shows that bursting is supported by activation of two different types of ion channels.

Analysis of ion channels responsible for the initiation of bursting and tonic firing and the transition between both is high physiological importance. A similar function of erg currents to induce bursting has recently been observed in dopaminergic subthalamic neurons (Huang et al., Sci. Adv. 2017;3: e1602272).

It is important to note that the type of bursting behaviour (clustered, high frequency of high amplitude action potentials on a depolarizing plateau) reported in the Huang et al (2017) paper is different from the pseudo-plateau bursting (maintained depolarization with potential smaller spikelets on plateau, as in Figure 4C and E) seen in endocrine cells such as corticotrophs. We have provided a short comment on the role of Erg in this neuronal bursting behaviour in the discussion (page 22) that complements the data we report in this type of bursting induced by CRH in corticotrophs.

Anyhow, this paper is unsatisfactory for several reasons:

1. The biophysical properties of erg currents recorded from male and female corticotrophs exhibit large differences. These differences are neither explained nor analyzed.
As detailed below in response to point 3, we see differences in biophysical properties of recorded Erg currents between individual corticotrophs of the same sex – both sexes display both fast and slow current kinetics as shown in Figure 2. We have edited the figure legend and results text to reflect this. We have further analysed the current kinetics in male and female corticotrophs as detailed in point 3 below. However, as the primary motivation of the paper was to examine the functional impact of Erg channels on corticotroph excitability we have not attempted to define or analyse these differences in greater detail. As outlined in point 3 below, we discuss a number of mechanisms that may explain this variability on page 20 but more detailed validation is outside the scope of this MS.

2. Since both, by which both channel types contribute to bursting are not further analyzed and discussed. The mathematical model described in the paper does not provide further analysis, especially it does not show any bursting and accommodation.

We are not clear what the reviewer means by both channels. In the results and discussion we highlight that our data reveal a role for Erg channels as well as the previously published role for BK channels in supporting CRH-induced bursting in corticotrophs. This is supported by our mathematical model including discussion in results and discussion sections.

We respectively disagree with the reviewer - the mathematical model provides new insight as we clearly demonstrate that inclusion of Erg conductances supports bursting in both the presence and absence of BKnear channels. Accommodation is mainly used to describe accommodation between spikes during a train of spikes as seen in a classical bursting behaviour in neurons. This is different from Pseudo-plateau bursting induced by CRH in corticotrophs (that lack multiple high amplitude spikes during the maintained depolarization) that we model in Figure 7 and thus analysis of potential accommodation during a burst is not relevant in this system.

The authors should consider the following points:

1. Figure 1 and page 10, line 5: The Kcne mRNA level of female corticotrophs is larger than that of male corticotrophs. Is it significantly larger? The possible effect of MIRP1 on erg currents should be discussed.

There is no statistically significant difference in Kcne mRNA level between male and female. We briefly discuss the potential role of MIRP1 on Erg currents in the results/discussion as per point 3 below.

2. Page 11: what is the meaning of "the proportion of peak biophysically isolated current" ?

The pulse protocol biophysically isolates a voltage dependent current characteristic of Erg currents. However, to verify this is an Erg current cells were exposed to E4031. The size of the current remaining after E4031 application was variable between cells and thus the proportion of the current that was sensitive to E4031was variable between cells. We have reworded this to provide further clarification.

3. Figure 2A, B illustrates the differences between erg currents recorded from male and female corticotrophs. Recorded at the same membrane potentials, the kinetics of male erg currents are much faster than female erg currents. At least, at the mRNA level there seems to be no difference. Presumably, at the protein level there is also no difference. The activation curves of male and female erg currents are also very similar. Nevertheless, the shape of the activation curves suggests that two different activation curves may exist, superimposed on each other. The authors should try to fit the activation data by two sigmoidal curves. In addition, the authors should evaluate the voltage dependence of the time constants. It will be very unsatisfactory, if there is no explanation for these strong differences. One solution to this problem could be to concentrate just on male or female corticotrophs.

In figure 2 we provide traces from both male and female corticotrophs and the traces used show currents with different kinetics. We used the traces to show the variability between individual corticotrophs however, as described in the corresponding results text the density and kinetics were different between individual cells, irrespective of whether they are male or female. As such males do not have faster deactivation kinetics than females – we see the same fast and slow current kinetics in both male and female cells. We now provide new data fitting the current traces, determined at -120 mV from a step of 40 mV, to a double exponential and reporting the time constants. As we reveal, currents with either the same fast or slow kinetics are observed in both male and female cells. We have edited the manuscript including the Figure legend to clarify this issue. However, as the key focus was the functional effect of Erg currents on corticotroph excitability we did not examine the kinetic properties in more detail.

The activation curves determined using the multi-step potential protocol in individual cells were best fit by a Boltzmann function with similar V50max and k values between males and females and reported in the revised manuscript. These values agree with previous data on Erg currents in native and recombinant systems in the elevated extracellular potassium recording conditions used here.

Multiple different mechanisms may underlie the observed differences in Erg current kinetics and amplitude between cells as we now include on page 20 of discussion. Firstly, different alternate transcripts, due to different start sites in the promoter, or alternative mRNA splicing of Kcnh2 can dramatically modify deactivation kinetics with variants disrupting the PAS-CNBHD interaction displaying much faster activation and deactivation without affecting V0.5 max [Sanchez-Conde 2022, Codding & Trudeau, 2019; Wang et al 1998]. In addition, co-expression with MIRP1 can reduce both Kcnh2 single channel conductance, accelerate deactivation and shift V0.5max in the positive direction [Abbott et al 1999]. Alternatively, while on average our RNA-seq analysis reveals that in the corticotroph population *on average* Kcnh2 is the dominant transcript in both males and females we do not know if at the single cell level the same ratios apply between cells. Thus, intrinsic differences between cells may also reflect different expression of Kcnh subunits between individual cells. For example, Kcnh7 homomultimers have much faster deactivation kinetics than Kcnh2 homomultimers (Sanchez-Conde 2021; Wimmer et al 2002).

While we agree that future studies would be beneficial to understand the molecular and or regulatory basis for these differences in Erg current kinetics at the single corticotroph level this is beyond the scope of the current manuscript. We feel that reporting that both male and female corticotrophs express functional Erg currents is important but more detailed analysis of the basis of variability in Erg current kinetics detracts from the novel insights into the functional role of Erg currents we define in corticotrophs.

We have included further analysis and discussion of these points in the methods, results and discussion

4. Page 12, lines 9 and 12: references should be added to statements taken from the literature. This is true for several other statements throughout the text which also need to be verified by references.

Thank you for highlighting this omission. We have now included references as appropriate and checked other instances.

5. Figure 3C: Although there was no change in V50max determined in control cells, was there any change in the shape of the activation curves of erg currents in the presence of CRH and AVP?

There was no significant change in shape of the activation curve after CRH+AVP and we now include changes in slope values in the text to highlight this. In males the change (delta) in slope after CRH+AVP was (0.03 +_ 2.18, n = 6) and in females (0.28 +_ 1.68, n = 5). This is now included in the revised manuscript

6. Figure 4: The letters are missing to identify the six panels.

Thank you for highlighting this oversight – this has been corrected

A, B: At least the recordings selected to represent basal conditions show a small depolarization in the recording of the E-4031-treated corticotroph. In addition, the number of

spikes doubled in the presence of E4031. Obviously, these recordings are not representative. On the other hand, such a depolarization is plausible, since the activation curves of Fig. 2D show a steady state erg current at the resting potential of -45 mV (female) and -50 mV (male).

Please note these basal traces are representative recordings but are not repeated measures from the same cell before and after E4031 due to the different treatments applied. For representative traces we randomly select a region from relevant traces that represents a fraction of the duration (3 minutes) over which parameters are determined. Please note that spontaneous action potential frequency of corticotrophs is low and highly variable – there was no significant effect of inhibiting Erg on spontaneous action potential frequency or membrane potential across cells as reported in the original MS. We have clarified text and legends to clarify this point.

C-F: These data are very nice and convincing. I suggest to indicate the level of the resting potential in all recordings. This would help to estimate the absolute value of the resting potential and the magnitude of the transient hyperpolarizations following the bursts.
We have now included a dotted line to indicate resting membrane potential

7. Page 13: The resting potential of female corticotrophs is about 5 mV more depolarized than that of male corticotrophs. Is there any explanation for this difference?

Although the mean value numerically differs by 5 mV in these particular experiments the RMP is not significantly different between male and female corticotrophs as we reported in the MS (page 14) due to the intrinsic variation and hence relatively large SD between cells. This is in accordance with our previous detailed analysis revealing no significant difference in RMP between male and female corticotrophs (see Duncan et al 2023). Male and female corticotrophs do display a number of differences in both spontaneous and evoked excitability although the molecular basis for this is yet to be defined. Resting membrane potential in corticotrophs is finely tuned by the dynamic balance between inward currents mediated by Nalcn as well as background potassium conductances [Belal et al 2025, J Physiol 603:301-317]. Whether differences in the density/properties of these conductances between males and females contributes to such differences is not known. Furthermore, RNA-seq analysis has revealed significant differences in the ion channel mRNA expression landscape between male and female murine corticotrophs [Duncan et al 2023]. Clearly these are important areas for future investigation.

8. Figure 5 and 6: Both figures are difficult to understand. One improvement could be to add the levels of statistical significance to the figures themselves. Another suggestion is to remove the dofetilide data (just for clarity), and to summarize them in the text.

We feel that inclusion of the dofetilide data is most appropriate for Figure 5 otherwise we would have to repeat control data and allow comparison with E4031 in the text. We have now included the respective p values included in Figure legends on the Figures themselves.

9. What is the rationale for studying dofetilide?

As we used a pharmacological approach to interrogate the functional role of Erg currents in corticotrophs we wanted to use another pharmacological inhibitor of Erg, in addition to E4031, to verify the key findings on bursting behaviour. This is important as although E4031 is widely used as a selective Erg inhibitor it does have off target effects. We have clarified in the text.

10. Figure 7: The labeling of the panels (A-F) as well as the time and potential axes are

missing. As in Figure 4, it would be helpful to indicate the level of the resting potential for each calculation.

We have now included letters for each panel. Scale bars for membrane potential and time were used in the original figure (in centre bottom of panel). We feel this approach, rather than using Y and X axes in each traces are more helpful. We have used a dotted line to indicate resting membrane potential as above.

C, D: In addition to the level of the resting potential the level of the plateau potentials should be indicated. Can the model generate bursts and accommodation during bursts?

The model clearly generates pseudo-plateau bursting as observed in panels C and D, that is the major effect of CRH on corticotroph excitability modelled in Figure 7. Pseudo-plateau bursting is different from the type of plateau bursting seen in many neurons where clusters of high frequency action potential are generated on top of a plateau depolarization. In contrast, most CRH-induced bursts in corticotrophs, and other pituitary endocrine cells, are defined as pseudo plateaus bursts that do not include high frequency full height spikes during the depolarization. Please note that as previously reported CRH induces bursts whereas AVP largely increases spike frequency. As such, analysis of spike accommodation is not appropriate for the types of burst generated by CRH as in the model in Figure 7.

References (in original MS, new refs included in revised manuscript)

Abbott GW, Sesti F, Splawski I, Buck ME, Lehmann MH, Timothy KW, Keating MT & Goldstein SAN. (1999) MIRP1 forms IKr potassium channels with HERG and is associated with cardiac arrythmias. Cell **97**, 175-187.

Codding SJ & Trudeau MC. (2019). The hERG potassium chnanel intrinsic ligand regulates N- and C-terminal intercarions and channel closure. J Gen Physiol **151**, 478-488.

Duncan PJ, Romano N, Nair SV, Murray JF, Le Tissier P & Shipston MJ. (2023). Sex differences in pituitary corticotroph excitability. *Front Physiol* **14,** 1205162.

Huang C-S, Wang G-H, Tai C-H, Hu C-C & Yang Y-C. (2017) Antiarrhythmics cure brain arrythmia: The imperativeness of subthalamic ERG K^+ channels on parkinsonian discharges. Sci Adv **3**, e1602272.

Sanchez-Conde FG, Jimenez-Vazquez EN, Auerbach DS & Jones DK. (2022). The ERG1 K(+) Channel and Its Role in Neuronal Health and Disease. *Front Mol Neurosci* **15,** 890368.

Wang J, Myers CD & Roberston GA. (1998). Dynamic control of deactivation gating by a soluble amino-terminal domain on HERG K^+ channels. J Gen Physiol **115**, 749-758.

Wimmers S, Bauer CK & Schwarz JR. (2002). Biophysical properties of heteromultimeric erg K^+ channels. Pflug Arch **445**, 423-440.

Dear Dr Shipston,

Re: JP-RP-2026-290793R1 "**Erg currents support electrical bursting in murine anterior pituitary corticotrophs**" by Sooraj V Nair, Nicola Romanò, Peter J Duncan, Paul Le Tissier, Richard Bertram, and Michael J Shipston

Thank you for submitting your manuscript to The Journal of Physiology. It has been assessed by a Reviewing Editor and by 2 expert referees and we are pleased to tell you that it is acceptable for publication following satisfactory revision.

The referee reports are copied at the end of this email.

Please address all the points raised and incorporate all requested revisions or explain in your Response to Referees why a change has not been made. We hope you will find the comments helpful and that you will be able to return your revised manuscript within 4 weeks. If you require longer than this, please contact journal staff: jp@physoc.org.

Your revised manuscript should be submitted online using the link in your Author Tasks Link Not Available. This link is accessible via your account as Corresponding Author; it is not available to your co-authors. If this presents a problem, please contact journal staff (jp@physoc.org). Image files from the previous version are retained on the system. Please ensure you replace or remove any files that are being revised.

If you do not wish to submit a revised version of your manuscript, you must inform our journal staff (jp@physoc.org) or reply to this email to request withdrawal. Please note that a manuscript must be formally withdrawn from the peer review process at one journal before it may be submitted to another journal.

TRANSPARENT PEER REVIEW POLICY: To improve the transparency of its peer review process The Journal of Physiology publishes online, as supporting information, the peer review history of all articles accepted for publication. Readers will have access to decision letters, including Editors' comments and referee reports, for each version of the manuscript, as well as any author responses to peer review comments. Referees can decide whether or not they wish to be named on the peer review history document.

ABSTRACT FIGURES: Authors are expected to use The Journal's premium BioRender account to create/redraw their Abstract Figures. Information on how to access this account is here: https://physoc.onlinelibrary.wiley.com/journal/14697793/biorender-access.

This will enable Authors to create and download high-resolution figures. If authors have used the free BioRender service, they can use the instructions provided in the link above to download a high-resolution version suitable for publication.

The link provided should only be used for the purposes of this submission. Authors will be charged for figures created on this account if they are not related to this manuscript submission.

LANGUAGE EDITING AND SUPPORT FOR PUBLICATION: If you would like help with English language editing, or other article preparation support, Wiley Editing Services offers expert help, including English Language Editing, as well as translation, manuscript formatting, and figure formatting at www.wileyauthors.com/eeo/preparation. You can also find resources for Preparing Your Article for general guidance about writing and preparing your manuscript at www.wileyauthors.com/eeo/prepresources.

RIGOUR AND REPRODUCIBILITY: We recommend authors consult The Journal's Rigour and Reproducibility requirements to ensure that their manuscript is suitable for publication: https://physoc.onlinelibrary.wiley.com/pb-assets/hub-assets/physoc/documents/TJP-Rigour-and-Reproducibility-Requirements.pdf

REVISION CHECKLIST:

Check that your Methods section conforms to journal policy: https://jp.msubmit.net/cgi-bin/main.plex?form_type=display_requirements#methods

Check that data presented conforms to the statistics policy: https://jp.msubmit.net/cgi-bin/main.plex?form_type=display_requirements#statistics

Upload a full Response to Referees file. To create your 'Response to Referees' copy all the reports, including any comments from the Senior and Reviewing Editors, into a Microsoft Word, or similar, file and respond to each point, using font or background colour to distinguish comments and responses and upload as the required file type.

Please upload two versions of your manuscript text: one with all relevant changes highlighted and one clean version with no changes tracked. The manuscript file should include all tables and figure legends, but each figure/graph should be uploaded as separate, high-resolution files. The journal is now integrated with Wiley's Image Checking service. For further details, see: https://www.wiley.com/en-us/network/publishing/research-publishing/trending-stories/upholding-image-integrity-wileys-image-screening-service

You may also upload:

- 'Potential Cover Art' for consideration as the issue's cover image
- Appropriate Supporting Information (Video, audio or data set: see https://jp.msubmit.net/cgi-bin/main.plex?form_type=display_requirements#supp).

We look forward to receiving your revised submission.

If you have any queries, please reply to this email and we will be pleased to advise.

Yours sincerely,

Peying Fong
Senior Editor
The Journal of Physiology

REQUIRED ITEMS

- Papers must comply with the Statistics Policy: https://jp.msubmit.net/cgi-bin/main.plex?form_type=display_requirements#statistics.

In summary:

- If n {less than or equal to} 30, all data points must be plotted in the figure in a way that reveals their range and distribution. A bar graph with data points overlaid, a box and whisker plot or a violin plot (preferably with data points included) are acceptable formats.

- If n > 30, then the entire raw dataset must be made available either as supporting information, or hosted on a not-for-profit repository, e.g. FigShare, with access details provided in the manuscript.

- 'n' clearly defined (e.g. x cells from y slices in z animals) in the Methods. Authors should be mindful of pseudoreplication.

- All relevant 'n' values must be clearly stated in the main text, figures and tables.

- The most appropriate summary statistic (e.g. mean or median and standard deviation) must be used. Standard Error of the Mean (SEM) alone is not permitted.

- Exact p values must be stated. Authors must not use 'greater than' or 'less than'. Exact p values must be stated to three significant figures even when 'no statistical significance' is claimed.

- Please includedean Abstract Figure legend. An appropriate figure legend, which should not exceed 150 words in length, should be included in the main manuscript file.

EDITOR COMMENTS

Reviewing Editor:

The authors have addressed the major concerns of the reviewers. However, as reviewer 1 pointed out, the manuscript would benefit from another grammar check and check for minor errors.

Below is a list of some of the minor issues that caught my eye. They are likely a few more, so please double check carefully.

Line 306 missing +/-

Line 323 V50max - is this the same as V0.5max (line 324)? I think these should be the same, V50max.

Line 387, line 388 "E-4031" should be E4031 for consistency.

Line 429-430: Two "(" with only one ")"

Also line 438-439, unpaired "("

Line 438: second "Hz" missing

Line 535: Erg 3 is italicized but Erg2 is not?

Line 550: should Kcnh or Kcne be italicized if they refer to subunits? Please double check the italics throughout this section of the discussion.

Line 605: patients have the KCNH2 gene, not Kcnh2. Line 602 and 621 should probably also be the human KCNH2 not Kcnh2.

Senior Editor:

Review of your revised manuscript is now complete. As the attached reports indicate, both Expert Referees greatly appreciate the effort invested in preparing this revision. At this time, there remain only minor points, highlighted in the Reviewing Editor's report, that require your attention. I look forward to receiving these corrections and thank you for contributing your work to The Journal of Physiology.

REFEREE COMMENTS

Referee #1:

The manuscript has improved after the changes. The authors have addressed all points. Limitations have been satisfactorily addressed in the discussion. Please do a grammar check and check for minor errors eg missing {plus minus} page 12.

Referee #2:

In the revised manuscript the authors have satisfactorily addressed all points raised in my previous review. The paper describes an ether-à-go-go-related gene K current in murine pituitary corticotrophs which contributes to their electrical bursting behavior.

The experimental observations are new and interesting. The experimental methods used are state-of-the art. In addition to

molecular biology and electrophysiology the authors employed modelling and - thereby - demonstrate the importance of erg K channels, and, as a second contributor, BK channels in modulating bursting behavior.

END OF COMMENTS

Response to referees & Editors

We thank the referees and Editors for their supportive comments on our revised manuscript. We are greatful to the reviewing editor for highlighting a number of minor typographical errors for correction.

We have addressed all these issues and carefully analysed the rest of the manuscript to remove any remaining minor typographical/grammatical errors.

On behalf of all authors, I hope that our manuscript is now suitable for publication in the Journal of Physiology.

Kindest regards

Mike Shipston

REQUIRED Items

- If n {less than or equal to} 30, all data points must be plotted in the figure in a way that reveals their range and distribution. A bar graph with data points overlaid, a box and whisker plot or a violin plot (preferably with data points included) are acceptable formats.

Data in figures are presented as box plots divided into quartiles and overlaid with data points from individual cells.

- If n > 30, then the entire raw dataset must be made available either as supporting information, or hosted on a not-for-profit repository, e.g. FigShare, with access details provided in the manuscript.

Access information for code and data included as appropriate, data do not exceed n > 30 in manuscript

- 'n' clearly defined (e.g. x cells from y slices in z animals) in the Methods. Authors should be mindful of pseudoreplication.
Included in methods

- All relevant 'n' values must be clearly stated in the main text, figures and tables.
Included throughout

- The most appropriate summary statistic (e.g. mean or median and standard deviation) must be used. Standard Error of the Mean (SEM) alone is not permitted.

Mean plus or minus standard deviation used throughout

- Exact p values must be stated. Authors must not use 'greater than' or 'less than'. Exact p values must be stated to three significant figures even when 'no statistical significance' is claimed.

Exact p values included throughout

- Please include an Abstract Figure legend. An appropriate figure legend, which should not exceed 150 words in length, should be included in the main manuscript file.

This is now included after the Figure legends in main manuscript

EDITOR COMMENTS

Reviewing Editor:

The authors have addressed the major concerns of the reviewers. However, as reviewer 1 pointed out, the manuscript would benefit from another grammar check and check for minor errors.

Below is a list of some of the minor issues that caught my eye. They are likely a few more, so please double check carefully.

COMMENTS

Line 306 missing +/-
Corrected

Line 323 V50max - is this the same as V0.5max (line 324)? I think these should be the same, V50max.
Corrected this should be V_{50max} throughout, we have checked all other instances

Line 387, line 388 "E-4031" should be E4031 for consistency.
Corrected

Line 429-430: Two "(" with only one ")"
Corrected

Also line 438-439, unpaired "("
Corrected

Line 438: second "Hz" missing
Corrected

Line 535: Erg 3 is italicized but Erg2 is not?
Erg2 is now also italicised as referring to mRNA

Line 550: should Kcnh or Kcne be italicized if they refer to subunits? Please double check the italics throughout this section of the discussion.
Thankyou, the Editor is correct that in this case we are referring to the protein subunits so Kcnh and Kcne should not be italicised. We have edited this section and made explicit when we are referring to mRNAs compared to protein subunits.

Line 605: patients have the KCNH2 gene, not Kcnh2. Line 602 and 621 should probably also be the human KCNH2 not Kcnh2.
We have edited to include human gene/protein format

Senior Editor:

Review of your revised manuscript is now complete. As the attached reports indicate, both Expert Referees greatly appreciate the effort invested in preparing this revision. At this time, there remain only minor points, highlighted in the Reviewing Editor's report, that require your attention. I look forward to receiving these corrections and thank you for contributing your work to The Journal of Physiology.

REFEREE COMMENTS

Referee #1:

The manuscript has improved after the changes. The authors have addressed all points. Limitations have been satisfactorily addressed in the discussion. Please do a grammar check and check for minor errors eg missing {plus minus} page 12.
Corrected, we have checked manuscript throughout

Referee #2:

In the revised manuscript the authors have satisfactorily addressed all points raised in my previous review. The paper describes an ether-à-go-go-related gene K current in murine pituitary corticotrophs which contributes to their electrical bursting behavior.

The experimental observations are new and interesting. The experimental methods used are state-of-the art. In addition to

molecular biology and electrophysiology the authors employed modelling and - thereby - demonstrate the importance of erg K channels, and, as a second contributor, BK channels in modulating bursting behavior.
We have carefully reviewed the manuscript and made additional minor typographical changes as required throughout

Dear Professor Shipston,

Re: JP-RP-2026-290793R2 "**Erg currents support electrical bursting in murine anterior pituitary corticotrophs**" by Sooraj V Nair, Nicola Romanò, Peter J Duncan, Paul Le Tissier, Richard Bertram, and Michael J Shipston

We are pleased to tell you that your paper has been accepted for publication in The Journal of Physiology.

The last Word (or similar) version of the manuscript provided will be used by the Production Editor to prepare your proof. When this is ready you will receive an email containing a link to Wiley's Online Proofing System. The proof should be thoroughly checked and corrected as promptly as possible.

Authors should note that it is too late at this point to offer corrections prior to proofreading. Major corrections at proof stage, such as changes to figures, will be referred to the Editors for approval before they can be incorporated. Only minor changes, such as to style and consistency, should be made at proof stage. Changes that need to be made after proof stage will usually require a formal correction notice.

All queries at proof stage should be sent to: TJP@wiley.com.

Following copyediting and typesetting the accepted version will be published online.

Yours sincerely,

Peying Fong
Senior Editor
The Journal of Physiology

IMPORTANT POINTS TO NOTE FOLLOWING ACCEPTANCE OF YOUR PAPER:

• IMPORTANT NOTICE ABOUT OPEN ACCESS: To assist authors whose funding agencies mandate immediate public access to published research findings, The Journal of Physiology allows authors to pay an Open Access (OA) fee to have their papers made freely available immediately on publication.

The Corresponding Author will receive an email from Wiley with details on how to register or log in to Wiley Authors where you will be able to place an order.

• You can check if your funder or institution has a Wiley Open Access Account here:
https://authors.wiley.com/author-resources/Journal-Authors/open-access/author-compliance-tool.html

• TRANSPARENT PEER REVIEW POLICY: To improve the transparency of its peer review process, The Journal of Physiology publishes online as supporting information the peer review history of all articles accepted for publication. Readers will have access to decision letters, including Editors' comments and referee reports, for each version of the manuscript as well as any author responses to peer review comments. Referees can decide whether or not they wish to be named on the peer review history document.

• You can help your research get the attention it deserves! Check out Wiley's free Promotion Guide for best-practice recommendations for promoting your work at: www.wileyauthors.com/eeo/guide. You can learn more about Wiley Editing Services which offers professional video, design, and writing services to create shareable video abstracts, infographics, conference posters, lay summaries, and research news stories for your research at: www.wileyauthors.com/eeo/promotion.

• If you would like to receive our 'Research Roundup', a monthly newsletter highlighting the cutting-edge research published in The Physiological Society's family of journals (The Journal of Physiology, Experimental Physiology, Physiological Reports, The Journal of Nutritional Physiology and The Journal of Precision Medicine: Health and Disease), please click this link, fill in your name and email address and select 'Research Roundup':
https://www.physoc.org/journals-and-media/membernews

EDITOR COMMENTS

Reviewing Editor:

The authors have complied with all suggestions and the manuscript has been appropriately revised.

Senior Editor:

Thank you for making all corrections requested in the previous round of review of your manuscript. Congratulations on a job well done!
